# Integrin β3 deficiency unleashes spontaneous pulmonary inflammation by promoting B cell hyperactivation via the CD40-CD40L axis

**DOI:** 10.3389/fimmu.2026.1796926

**Published:** 2026-03-24

**Authors:** Shuang Liu, Kaiwen Wang, Jiayi Chen, Yan Shen, Zheng Ruan, Kankan Wang, Xiaodong Xi, Jianhua Mao

**Affiliations:** 1 Shanghai Institute of Hematology, State Key Laboratory of Medical Genomics, National Research Center for Translational Medicine at Shanghai, Collaborative Innovation Center of Hematology, Ruijin Hospital, Shanghai Jiao Tong University School of Medicine, Shanghai, China; 2 Research Center for Experimental Medicine, Ruijin Hospital, Shanghai Jiao Tong University School of Medicine, Shanghai, China; 3 Shanghai Key Laboratory of Gene Editing and Cell-based Immunotherapy for Hematological Diseases, Ruijin Hospital, Shanghai Jiao Tong University School of Medicine, Shanghai, China; 4 Sino-French Research Center for Life Sciences and Genomics, Ruijin Hospital, Shanghai Jiao Tong University School of Medicine, Shanghai, China

**Keywords:** autoimmune disease, B cell activation, CD40-CD40L axis, immune homeostasis, integrin β3, spontaneous pulmonary inflammation

## Abstract

**Background:**

Pulmonary immune homeostasis requires tight control of adaptive responses. Integrin β3 is a well-known mediator of cell adhesion and platelet function. However, its role in adaptive immunity, especially in B cell responses, remains unclear.

**Methods:**

We defined the pulmonary phenotype of constitutive β3-deficient (β3^−/−^) mice by histopathology. We performed integrated transcriptomic and proteomic profiling of lung tissue to map the molecular signature of spontaneous pulmonary inflammation. We further probed the underlying mechanisms with additional histology and functional assays and tested for biological significance using transcriptomics data from auto-immune disease patients.

**Results:**

β3^−/−^ mice developed spontaneous pulmonary inflammation marked by B cell activation and *in situ* immune-complex deposition within alveoli. Multi-omics integration implicated the CD40-CD40 Ligand (CD40L) axis as a central driver of this pathology. Mechanistically, loss of β3 enhanced CD40L–CD40 engagement on B cells, resulting in NF-κB pathway hyperactivation. Consistent with our murine data, reduced *ITGB3* expression in patients with autoimmune disease correlated with transcriptional signatures of B cell activation and inflammation.

**Conclusions:**

These results reframe integrin β3 as a threshold regulator of B cell activation. The β3–CD40L–CD40 axis therefore represents a potential therapeutic target for B cell–mediated autoimmune diseases.

## 1 Introduction

As one of the largest interfaces between the body and the external environment, the lung presents an extensive surface area that remains continuously exposed to diverse endogenous and exogenous stimuli as well as environmental challenges. Maintenance of normal pulmonary function therefore depends on a tightly regulated and dynamic process termed immune homeostasis (1). This balance relies not only on structural defenses, including physical barriers and the mucociliary clearance system, but also on coordinated interactions between innate and adaptive immune responses within the pulmonary microenvironment (2). Pulmonary immune homeostasis is naturally delicate, and breakdown in the pro-inflammatory and anti-inflammatory signaling often leads to chronic or spontaneous inflammation (3). While early studies emphasized the contribution of myeloid cells such as neutrophils and macrophages, recent reports suggest that B lymphocytes and other adaptive immunity pathways also play an important role in the maintenance of lung equilibrium and the regulation of natural progression of inflammatory disease within lungs (4).

Integrins are heterodimeric transmembrane receptors that mediate the interaction of cells with extracellular matrix (ECM) and transduce intracellular signals (5). The integrin β3 subunit, which pairs with αIIb or αv to form αIIbβ3 and αvβ3, is most notable for its essential roles in platelet activation, aggregation, angiogenesis, bone metabolism, and tumor metastasis (6). However, increasing evidence shows that β3 and its downstream signaling pathways also have important functions for inflammation and immune regulation (7, 8). Interestingly, αvβ3 has also been implicated in the regulation of spontaneous pulmonary inflammation via effects on macrophages, fibroblasts, and cytokine production (9). Despite these advances, the contribution of αvβ3 to adaptive immunity, particularly regarding B cells, remains incompletely understood.

Traditionally viewed as the principal effectors of humoral immunity, B cells primarily generate antigen-specific antibodies. Recent studies, however, have substantially broadened this view by demonstrating that B cells also play essential regulatory roles in spontaneous inflammation and immune homeostasis (10). In autoimmune diseases such as systemic lupus erythematosus (SLE), rheumatoid arthritis (RA), and RA–associated interstitial lung disease (RA-ILD), breakdown of immune tolerance promotes the formation of ectopic germinal centers and *in situ* immune complex deposition (11, 12). This pathological activation of B cells depends largely on interactions between CD40 expressed on B cells and CD40 Ligand (CD40L) expressed on T cells (13). Engagement of CD40 signaling activates downstream NF-κB and MAPK pathways, which drive B cell proliferation, differentiation, survival, and class-switch recombination within germinal centers, supporting the establishment of long-term immunological memory (14, 15). Importantly, dysregulated CD40–CD40L signaling represents a central mechanism underlying B cell–mediated spontaneous inflammation, as it disrupts immune tolerance and induces aberrant immune responses (14).

Following activation, a subset of B cells differentiates into antibody-secreting plasma cells. The antibodies produced by B cells can form antigen–antibody immune complexes (ICs), leading to activation of the complement cascade (16). Persistent formation and deposition of pathogenic ICs can initiate sustained inflammatory responses and progressive tissue damage (17). In addition, B cells actively modulate immune responses by upregulating co-stimulatory molecules through CD40 signaling, functioning as antigen-presenting cells, and secreting a broad range of cytokines (18). Collectively, these functions underscore the necessity of tightly regulated B cell activation and homeostasis for the maintenance of overall immune equilibrium.

Although the role of αvβ3 in innate immune cells is well established (19, 20), whether and how this integrin integrates intracellular signaling to regulate B cell activation, antibody production, and IC–mediated pathology remains unclear. Defining the contribution of αvβ3 to B cell–driven pulmonary and systemic immune homeostasis and inflammation is therefore essential to understand how immune balance shifts from physiological regulation to pathological inflammation. Elucidating these integrin-mediated mechanisms is particularly important because they may uncover new therapeutic targets for B cell–mediated lung diseases, which are currently managed largely with nonspecific immunosuppressive strategies.

To fill this knowledge gap, we examined the role of integrin αvβ3 in B cell–mediated immune homeostasis and spontaneous inflammation. We employed combined transcriptomic and proteomic profiling in β3deficient (β3^−/−^) mice to define the inflammatory landscape of the lung. Subsequently, we performed functional and mechanistic analyses to determine how αvβ3 regulates B cell activation thresholds and effector functions, with particular emphasis on the CD40–CD40L signaling axis. Finally, to establish clinical relevance, we analyzed transcriptomic datasets from patients with autoimmune diseases to associate reduced *ITGB3* expression with enhanced B cell activation signatures and to evaluate its potential diagnostic value. Together, this study delineates an αvβ3 integrin–B cell inflammation axis, redefines this integrin as a key immunoregulatory molecule, and identifies potential therapeutic targets for antibody-mediated pulmonary pathologies.

## 2 Material and methods

### 2.1 Mice

C57BL/6 mice were purchased from Shanghai Lingchang Biotechnology Co., Ltd. β3^−/−^ mice (C57BL/6 background) were kindly given by Professor Junling Liu from Shanghai Jiao Tong University School of Medicine (SHSMU). All experimental mice (8–10 weeks old) were housed under specific pathogen-free (SPF) conditions at the SHSMU animal facility. Mice were anesthetized with 2% isoflurane before all experimental procedures. The Animal Ethical Committee of SHSMU approved all animal experiments.

### 2.2 Western blot

Lung tissues obtained from WT and β3^−/−^ mice were rinsed with ice-cold PBS and lysed using RIPA with protease inhibitor cocktail and phosphatase inhibitor cocktail. Lysates were clarified by centrifugation at 4 °C and protein concentrations were measured using a BCA Protein Assay Kit.

Equal amounts of protein (10 μg per lane) were separated by 10% SDS-PAGE and subsequently transferred onto PVDF membranes. The membranes were incubated overnight at 4 °C with primary antibodies against integrin β3, CD40L, IκBα, phosphorylated IκBα (p-IκBα), NF-κB p65, phosphorylated NF-κB p65 (p-NF-κB p65), and GAPDH. After incubation with HRP-conjugated secondary antibodies, immunoreactive bands were detected using a digital imaging system.

### 2.3 RNA sequencing and transcriptomic analysis

Lung and spleen tissues were harvested from the same individual WT and β3^−/−^ mice (n = 3). Total RNA was isolated using TRIzol, and genomic DNA was removed by DNase I treatment. RNA integrity was confirmed with an Agilent Bioanalyzer (RIN > 7.0), and RNA was quantified using a NanoDrop spectrophotometer. Libraries were sequenced on an Illumina NovaSeq 6000 platform.

Raw sequencing reads were quality-trimmed using Trimmomatic and aligned to the mouse reference genome (GRCm39) with HISAT2. Gene-level reading counts were generated using featureCounts. Differential expression analysis was performed using DESeq2, with genes considered significantly differentially expressed if |log_2_ fold change| > 1 and adjusted *P* < 0.05. GO and KEGG pathway analyses were performed using clusterProfiler (v3.14.3).

### 2.4 Proteomics analysis

Lung and spleen tissues were collected from WT and β3^−/−^ mice (*n* = 3), snap-froze them in liquid nitrogen, and stored them at −80 °C. Proteins were extracted using urea-based lysis buffer, and protein concentration was determined by BCA assay.

Peptides were separated on a reversed-phase column (25 cm × 100 μm) using an EASY-nLC 1200 system with a 30 min gradient and analyzed by Orbitrap Exploris 480 mass spectrometer in data-independent acquisition (DIA) mode. Full MS scans (450–850 m/z) were acquired at 30,000 resolution, and MS/MS scans used HCD fragmentation at 28% NCE. Raw data were processed with DIA-NN (v1.8) against the *Mus musculus* UniProt database. Search parameters included trypsin/P digestion, one missed cleavage, fixed modifications, and 1% FDR.

Differentially expressed proteins were identified using a fold change > 1.5 and *P* < 0.05 criteria. Functional annotation and enrichment analyses (GO, KEGG, Reactome) were performed using Fisher’s exact test with P < 0.05.

### 2.5 Histological and immunohistochemical analysis

The lung tissues from WT and β3^−/−^ mice were fixed in 4% paraformaldehyde for 24 h, followed by dehydration and paraffin embedding. For histological evaluation, 4-μm-thick sections were prepared and stained with Hematoxylin and Eosin (HE) to evaluate tissue architecture pathological alterations, and inflammatory cell infiltration.

For IHC staining, sections were deparaffinized and rehydrated through a graded series of ethanol. Endogenous peroxidase activity was quenched with 3% H_2_O_2_ for 15 min and nonspecific binding was blocked with 5% normal goat serum. Sections were then incubated sections overnight at 4 °C with primary antibodies against CD45, CD3, CD68, CD20, CD19, CD40, and C3. After incubation with HRP-conjugated secondary antibodies, a DAB (3,3’-diaminobenzidine) kit was used for visualization. The slides were counterstained with hematoxylin, dehydrated, and mounted. Images were captured using a light microscope (Leica, Germany). Histological quantification was performed using the ImageJ software.

### 2.6 Immunofluorescence analysis

Frozen sections were prepared from tissues embedded in optimal cutting temperature (OCT) compound. For the first panel, sections were co-stained with antibodies against IgG and C3 to evaluate immune complex deposition. For the second panel, sections were incubated with antibodies against CD40L and CD19. The slides were incubated with appropriate secondary antibodies conjugated with Alexa Fluor-488 or Alexa Fluor-594. Nuclei were counterstained with DAPI for 5 min. The sections were imaged using confocal fluorescence microscopy (Leica, Germany).

### 2.7 Flow cytometry

Single−cell suspensions were prepared from freshly harvested lung tissues. Tissues were minced and enzymatically digested with collagenase IV (1mg/mL) and DNase I (50 μg/mL) at 37 °C. Digested samples were filtered through a cell strainer (40 μm) to generate a single−cell suspension, and residual red blood cells were lysed using ACK lysis buffer. For surface staining, the single-cell suspensions were aliquoted into separate tubes for parallel analyses. Each aliquot was stained with a common backbone panel comprising antibodies against CD45 and CD19 to identify B cell populations. Then, each aliquot was stained with one of the following specific target antibodies: anti-IgM, anti-IgG1, anti-CD80, or anti-PD-L2. All staining procedures were performed according to the manufacturer’s instructions. Flow cytometric analysis was performed using a flow cytometer, and data were analyzed using FlowJo software.

### 2.8 Data acquisition and processing

We downloaded transcriptomic datasets from the Gene Expression Omnibus (GEO) under accession numbers GSE50772, GSE93272, and GSE50635. Raw data were downloaded using the GEOquery R package. Probes were annotated to gene symbols using the corresponding platform annotation files. For genes represented by multiple probes, the average expression value was calculated.

### 2.9 Pan-cancer expression analysis

TIMER 3.0 and Gene Expression Profiling Interactive Analysis (GEPIA) online platforms were used for analyzing gene expressions in various cancers. The Cancer Genome Atlas (TCGA) database and Genotype-Tissue Expression (GTEx) database were used to compare the expression levels of *ITGB3* in cancer and normal tissues.

### 2.10 Immune cell infiltration analysis

Immune cell infiltration was evaluated by two complimentary computational methods. We first screened the association of *ITGB3* expression with immune cell infiltration at the pan-cancer level, particularly for the B cell lineage, using the TIMER3.0 database. We then estimated the absolute fraction of immune cells within every autoimmune disease cohort using the Microenvironment Cell Populations-counter (MCP counter) R package.

### 2.11 Diagnostic value and correlation analysis

The diagnostic value of *ITGB3* in distinguishing patients from healthy control groups was analyzed using the ROC curves through the pROC R package, and AUCs were determined as a measure of prediction power. Spearman’s correlation test was performed to assess correlations of *ITGB3* expressions with B cell activation biomarkers, including *CD40LG* and *MS4A1.*

### 2.12 Statistical analysis

Data were presented as mean ± SD. Comparisons were done by two-tailed unpaired Student’s *t*-test, for normally distributed data with equal variance assumed (GraphPad Prism 9). P < 0.05 was considered statistically significant. Data points are shown individually for visualization of spread.

## 3 Results

### 3.1 Deficiency of β3 integrin leads to spontaneous pulmonary inflammation

**Figure 1:**
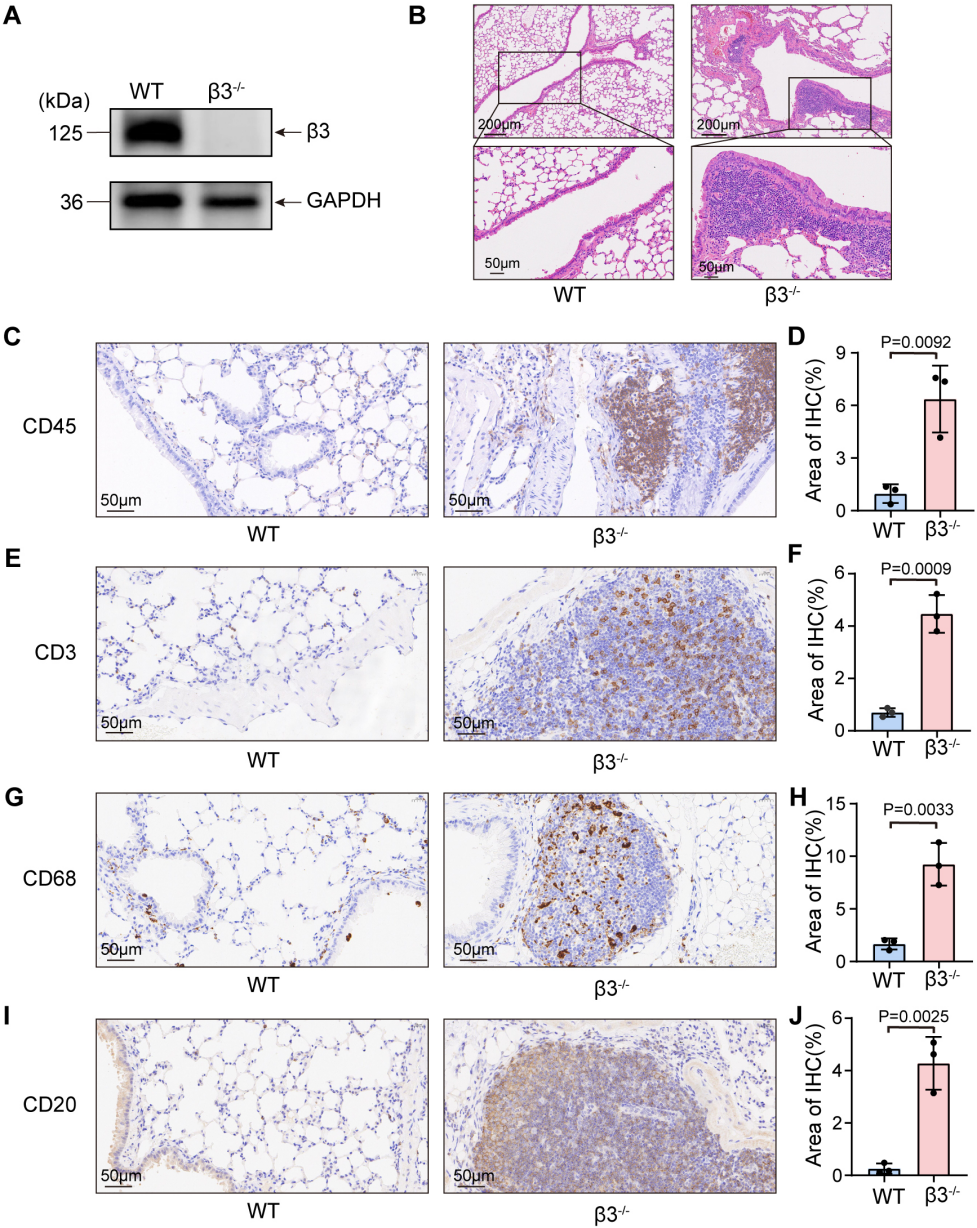
Genetic ablation of integrin β3 elicits spontaneous pulmonary inflammation. **(A)** Western blot analysis of β3 integrin expression in lung tissue lysates from WT and β3^−/−^ mice. GAPDH is used as a control. **(B)** Representative hematoxylin and eosin (HE) staining of lung sections from WT and β3^−/−^ mice. **(C–J)** Representative immunohistochemical (IHC) images and quantification of immune cell infiltrates, including CD45 **(C, D)** (total leukocytes), CD3 **(E, F)** (T cells), CD68 **(G, H)** (macrophages), and CD20 **(I, J)** (B cells) in lung sections from WT and β3^−/−^ mice (n = 3). Scale bar, 50 μm. Data are presented as mean ± SD. Statistical significance is determined using a two-tailed Student’s *t*-test (GraphPad Prism).

A constitutive β3^−/−^ mouse model was used to assess the role of integrin β3 in maintaining pulmonary immune homeostasis. Although these mice are well-characterized for susceptibility to Glanzmann thrombasthenia-like bleeding disorders and osteosclerosis due to platelet and osteoclast dysfunction (21, 22), their susceptibility to pulmonary immune dysregulation remains less defined. Western blot analysis of lung tissue lysates confirmed complete loss of β3 protein expression in knockout mice, validating the integrity and reliability of the β3^−/−^ model (**Figure 1A**). Histopathological examination revealed a clear spontaneous inflammatory phenotype in the lungs of β3^−/−^ mice compared with WT controls. This phenotype was characterized by focal accumulation and infiltration of immune cells, primarily lymphocytes and macrophages, localized to the perivascular and interstitial compartments (**Figure 1B**; **Supplementary Figure 1**). This process was accompanied by mild alveolar septal thickening (**Figure 1B**; **Supplementary Figure 1**). Collectively, these changes indicate a spontaneous disruption of normal pulmonary parenchymal architecture.

IHC analysis further characterized the inflammatory infiltrates and demonstrated a significant increase in total CD45^+^ leukocytes in the lungs of β3^−/−^ mice (**Figures 1C, D**). Although we observed elevated numbers of CD3^+^ T cells (**Figures 1E, F**) and CD68^+^ macrophages (**Figures 1G, H**), CD20^+^ B cells showed a striking and preferential accumulation (**Figures 1I, J**). This selective enrichment of B cells suggests a shift of the pulmonary immune microenvironment toward a humoral immune phenotype, providing a strong rationale for subsequent investigation of the molecular mechanisms driving B cell–biased inflammation.

### 3.2 Transcriptomic profiling implicates B cell receptor and inflammatory signaling in β3-deficient lungs

**Figure 2:**
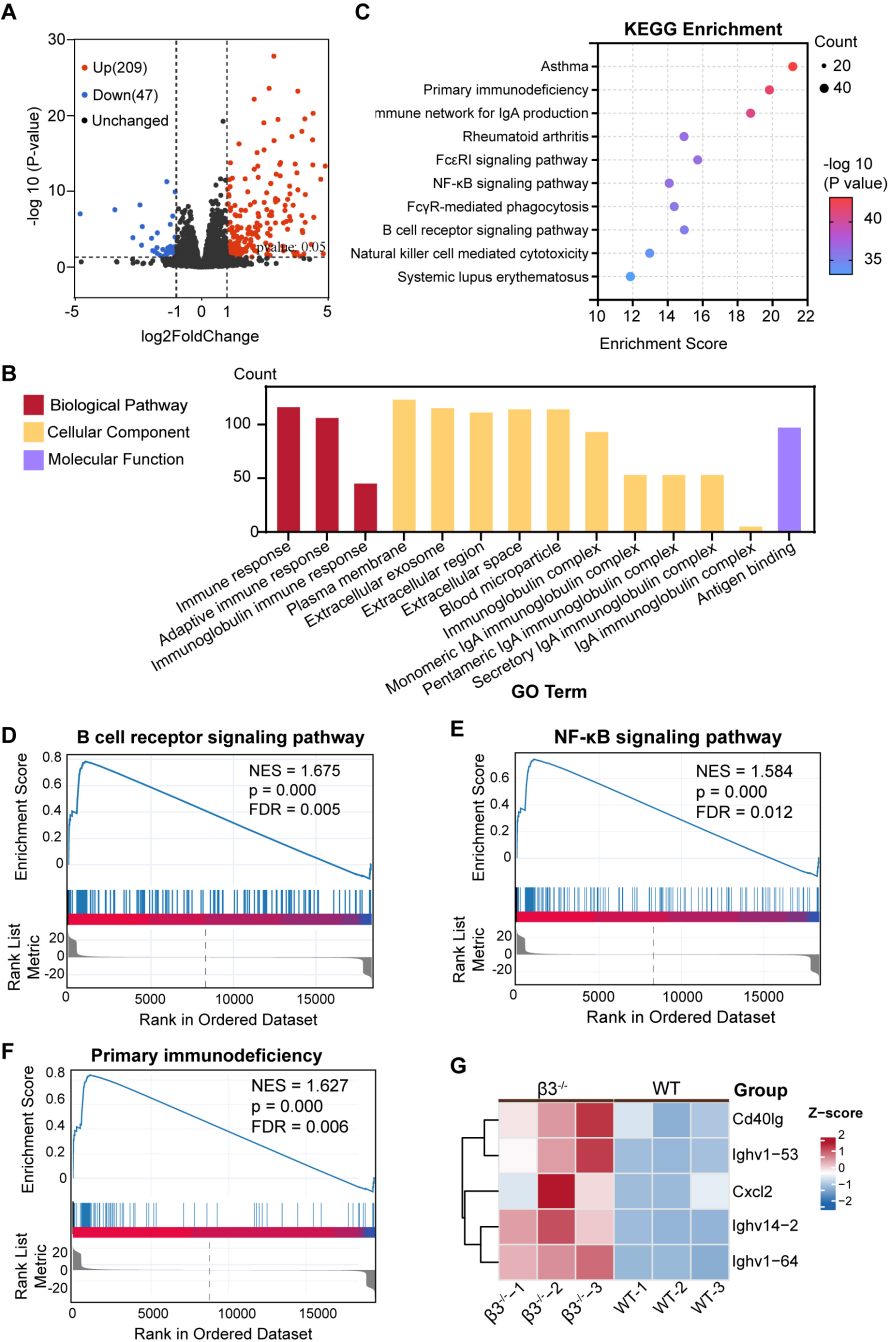
Transcriptomic profiling reveals aberrant B cell receptor and inflammatory signaling in β3^−/−^ lungs. Data were obtained from RNA-seq analysis of lung tissues from WT and β3^−/−^ mice (n = 3). **(A)** Volcano plot of differentially expressed genes (DEGs) in β3^−/−^ lungs compared to WT from RNA-seq. Red dots represent upregulated genes (log2FC > 1, P-adj < 0.05), and blue dots represent downregulated genes (log2FC < -1, P-adj < 0.05). **(B)** GO enrichment analysis of biological processes associated with the DEGs. Significantly enriched GO terms are shown. **(C)** KEGG pathway analysis of DEGs from RNA-seq. Each pathway is represented by a bubble; bubble size reflects the number of DEGs per pathway, and colors represent enrichment significance (-log10(P-value)). **(D–F)** Gene Set Enrichment Analysis (GSEA) of KEGG pathways. Enrichment plots for the significantly enriched pathways, **(D)** B cell receptor signaling pathway, **(E)** NF-κB signaling pathway, and **(F)** Primary immunodeficiency. For each plot, the running enrichment score (ES) is plotted against the gene list ranked by the signal-to-noise ratio (β3^−/−^ vs. WT). The normalized enrichment score (NES), P value (*p*), and false discovery rate (FDR) q value are indicated. Vertical ticks in the middle portion denote genes contributing to the leading-edge (core enriched) subset. **(G)** Heatmap showing the expression levels of representative genes belonging to the enriched B cell receptor signaling pathway across WT and β3^−/−^ lung tissue. Rows are genes, columns are biological replicates. Expression values are row-scaled Z-scores.

To define the signaling networks driving this spontaneous inflammatory response, we performed unbiased RNA sequencing of lung tissues. Differential expression analysis identified 256 differentially expressed genes (DEGs) in β3^−/−^ lungs, including 209 upregulated and 47 downregulated genes (**Figure 2A**). GO enrichment analysis showed that these DEGs were highly enriched in terms associated with the adaptive immune response, immunoglobulin complexes, and B cell activation (**Figure 2B**).

Kyoto Encyclopedia of Genes and Genomes (KEGG) pathway analysis further supported these findings which showed that immune-related signaling pathways were enriched in a significant manner, particularly the B cell receptor signaling pathway and primary immunodeficiency pathways (**Figure 2C**). KEGG analysis further revealed strong enrichment for inflammatory signaling cascades, the NF-κB signaling pathway, as well as pathways related to autoimmune diseases, like SLE and RA (**Figure 2C**). To provide quantitative evidence for these changes of pathways, we performed Gene Set Enrichment Analysis (GSEA). GSEA confirmed significant positive enrichment of three key pathways highlighted by the initial analyses: the B cell receptor signaling pathway (**Figure 2D**), the NF-κB signaling pathway (**Figure 2E**), and primary immunodeficiency (**Figure 2F**). In agreement with the KEGG results, we found that gene sets corresponding to certain human autoimmune and inflammatory disorders were also significantly activated within β3^−/−^ lungs (**Supplementary Figure 2**).

To visually identify co-regulated changes in B cell–associated signaling, we generated a heatmap using representative genes within the enriched B cell receptor (BCR) signaling pathway and found that there was an overall consistent increase across each β3^−/−^ biological replicate, including important signaling elements such as *Cd40lg* (CD40 ligand), along with other regulators (**Figure 2G**).Overall, these findings show that in the context of β3^−/−^ lungs, there is an up-regulation of genes related to activated B cells, increased inflammatory signaling, and pathways associated with human autoimmune disease.

### 3.3 CD40-CD40L axis is a mediator of pulmonary inflammatory pathology

**Figure 3:**
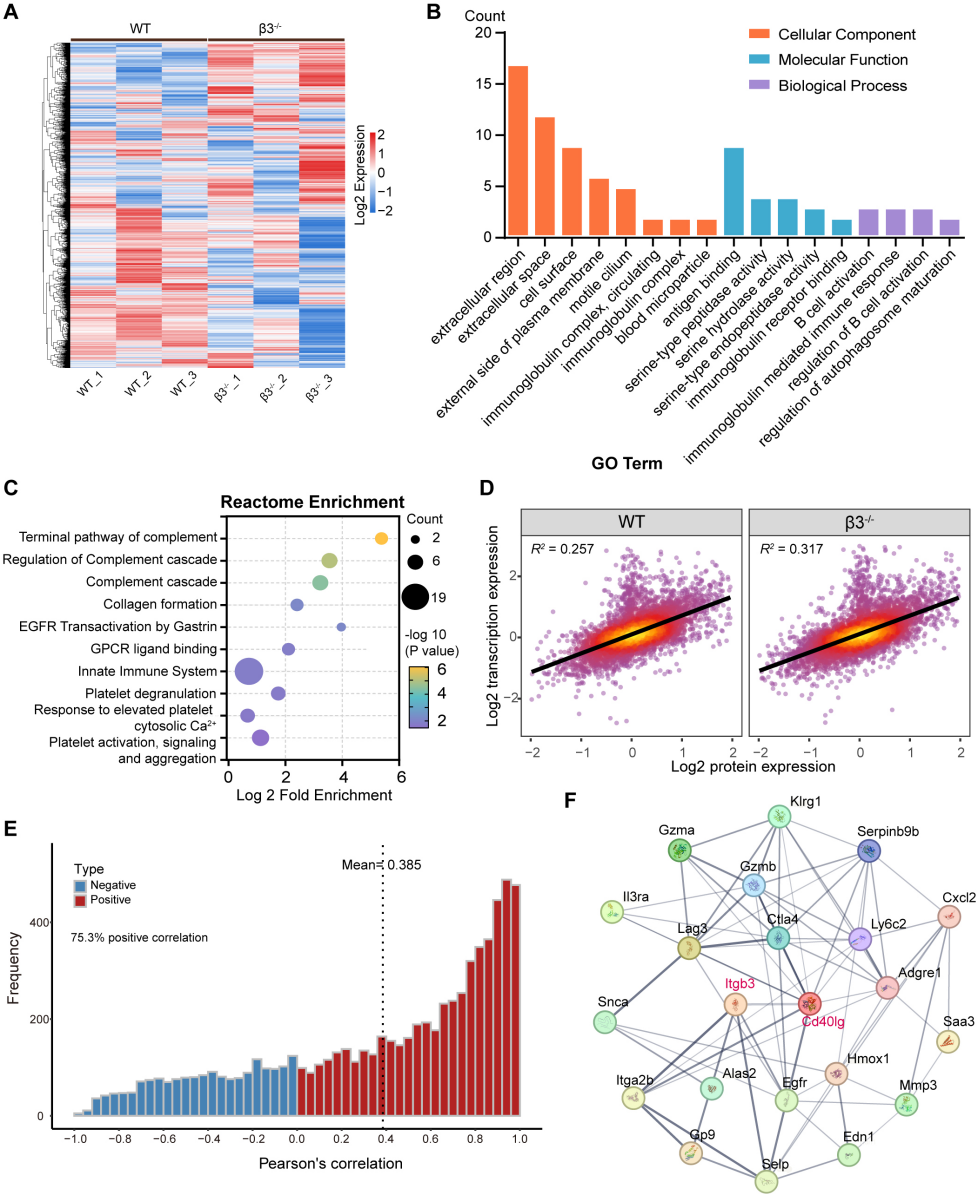
Proteomic profiling and integrated transcriptomic-proteomic analysis of β3^−/−^ lung tissues. Data represent proteomic analysis of lung tissues from WT and β3^−/−^ mice (n = 3). **(A)** Unsupervised hierarchical clustering heatmap of all quantified proteins across WT and β3^−/−^ lung samples. Rows represent individual proteins, and columns represent biological replicates. Protein abundance values are depicted as row-scaled Z-scores, with red indicating higher abundance and blue indicating lower abundance relative to the mean. **(B)** GO enrichment analysis of biological processes associated with the differentially abundant proteins (DAPs) identified in β3^−/−^ versus WT lungs. Significantly enriched GO terms are shown. **(C)** Reactome enrichment analysis of DAPs. Each pathway is represented by a bubble; bubble size reflects the number of differentially expressed proteins per pathway, and color intensity represents the -log10(adjusted P-value). **(D)** Scatter plot comparing transcriptomic (RNA-seq log2 fold change) and proteomic (protein abundance log2 fold change) alterations for matched genes/proteins between β3^−/−^ and WT lungs. The coefficient of determination (R²) from linear regression is indicated. **(E)** Cumulative distribution plots of Pearson correlation coefficients between matched transcript and protein expression levels across all samples within each genotype. The x-axis represents the Pearson correlation coefficient **(r)**, and the y-axis represents the cumulative proportion of gene-protein pairs. Curves are colored based on the direction of correlation: red for positive correlations (r > 0) and blue for negative correlations (r < 0). **(F)** Protein-protein interaction (PPI) network derived from a union of the DEGs and DAPs. The network was constructed using the STRING database.

We next tested for functional consequences of these transcriptional changes, by undertaking a quantitative proteomic screen. Unsupervised hierarchical clustering of all quantified proteins clearly separated β3^−/−^ samples from wild type controls, suggesting extensive proteomic reprogramming of β3^−/−^ lungs (**Figure 3A**). Consistent with the transcriptome data, we also observed that proteomic pathways were significantly altered. GO analysis revealed significant enrichment for terms associated with immunoglobulin complex, B cell activation and humoral immune response, and complement activation (**Figure 3B**). Consistently, Reactome pathway analysis showed strong complement related pathway activation, including the “Complement cascade,” “Terminal pathway of complement,” and “Regulation of complement cascade” (**Figure 3C**), which supported the existence of immune complex–mediated inflammatory pathology.

Next we performed integrative multi-omics analyses to investigate the concordance of transcriptional and proteomic changes. Scatterplots comparing mRNA with protein abundance revealed a positive linear correlation, suggesting a high level of biological correspondence across both data sets (**Figure 3D**). To quantify such correspondence, we used the cumulative distributions over the Pearson’s correlations, which resulted in a global positive shift for β3^−/−^ mice (**Figure 3E**).

To identify possible upstream regulators, we built a protein-protein interaction (PPI) network. In this network, we found that *Cd40lg* (CD40 Ligand) was a hub (**Figure 3F**). Because the CD40-CD40L axis has been implicated in B cell activation and immunoglobulin class switching (23), these data indicate that aberrant costimulation contributes directly to the observed inflammation phenotype.

### 3.4 β3 deficiency triggers humoral immune dysregulation in the spleen

**Figure 4:**
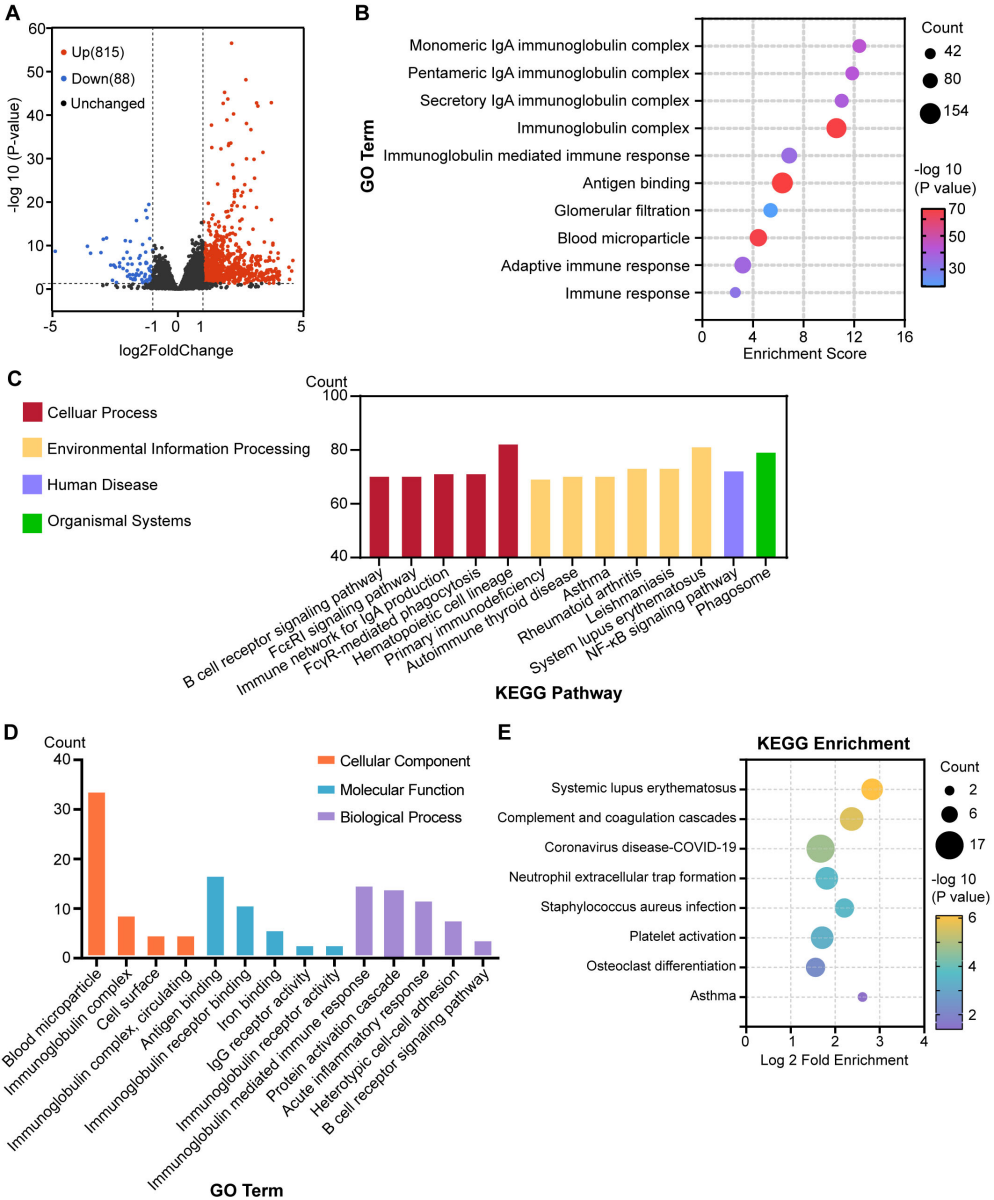
Multi-omics landscape of the spleen reveals systemic immune perturbations. **(A)** Volcano plot of transcriptomic changes in splenic tissues from WT and β3^−/−^ mice. **(B)** GO enrichment analysis of biological processes associated with the DEGs in splenic tissues. Significantly enriched GO terms are shown. **(C)** KEGG pathway analysis of DEGs from RNA-seq in splenic tissues from WT and β3^−/−^ mice. Each pathway is represented by a bubble; bubble size reflects the number of DEGs per pathway. **(D)** GO enrichment analysis of biological processes associated with the DAPs in splenic tissues. Significantly enriched GO terms are shown. **(E)** KEGG enrichment analyses of splenic DAPs. Each pathway is represented by a bubble. Bubble sizes reflect numbers of DEGs per pathway. Color intensity represents the -log10*P* value.

To determine if these immune defects were localized to the lung or indicative of an overall immunological deficit, we carried out concurrent transcriptomic and proteomic profiling of the spleen harvested from the same individual mice used for lung analysis. Although inherent tissue-specific differences exist between the baseline molecular profiles of lung and spleen, a comparative assessment revealed a striking conservation of inflammatory signatures. Transcriptomic analysis of β3^−/−^ spleens revealed the multiple pathways identified in the lung, specifically those for the immunoglobulin complex, adaptive immune response, and B cell receptor signaling pathway (**Figures 4A–C**). Similarly, proteomic profiling showed dysregulated proteins involved in the immunoglobulin complex (**Figures 4D, E**).

We further validated these systemic alterations through integrated multi-omics analysis of splenic tissues. Quantitative correlation analysis demonstrated a positive linear relationship between transcript and protein abundance (**Supplementary Figure 3A**), which was confirmed by cumulative distribution analysis of Pearson correlation coefficients (**Supplementary Figure 3B**). Taken together, these results suggest that β3 deficiency induces systemic immunological changes leading to the alteration of the signal transduction pathways in lymphocytes from different tissues and their consequent activation status.

### 3.5 Pulmonary B cell hyperactivation is associated with immune complex deposition and upregulated CD40-CD40L signaling

**Figure 5:**
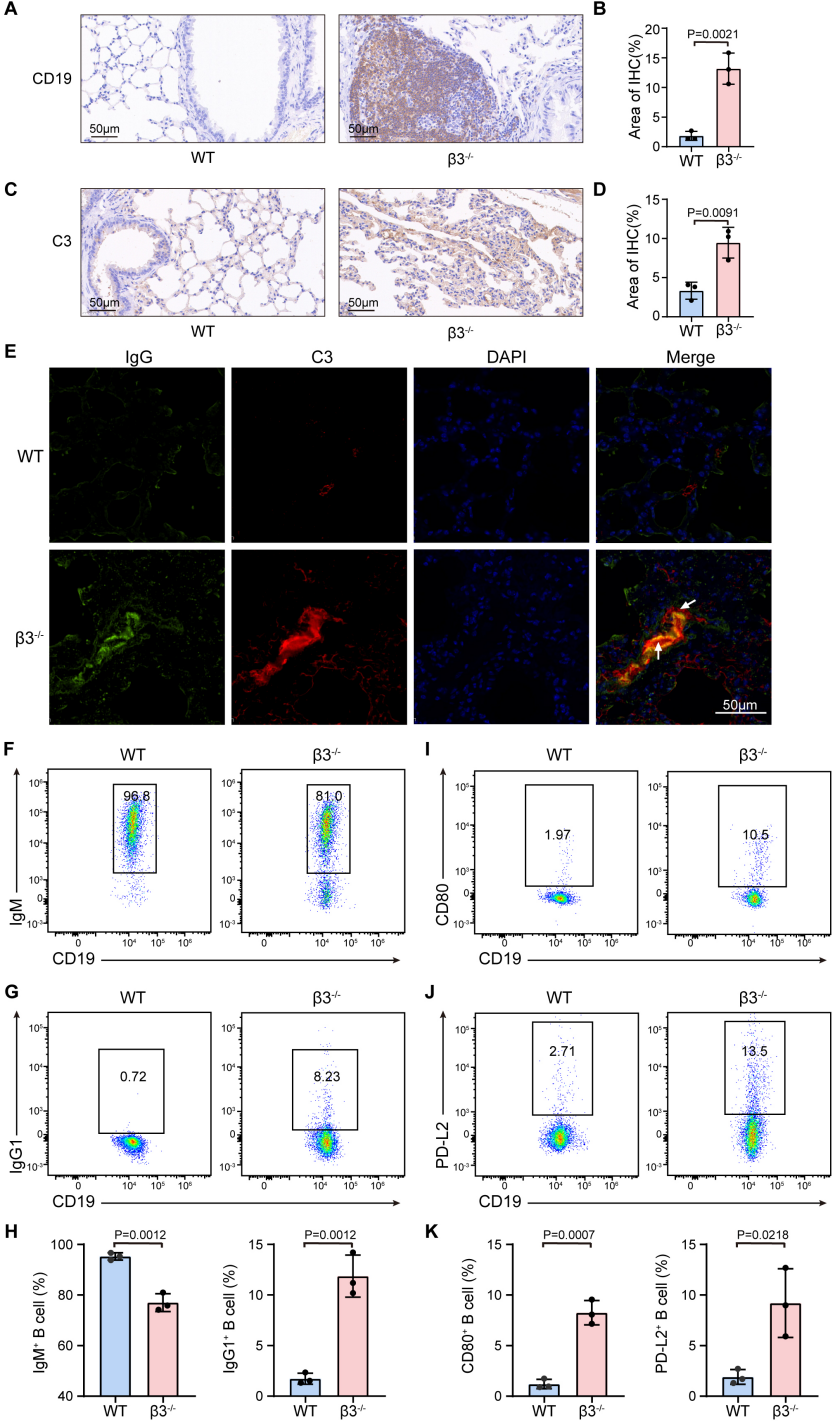
Pulmonary B cell expansion is associated with *in situ* immune complex formation and complement activation. **(A)** Representative IHC staining for CD19 in lung sections from WT and β3^−/−^ mice. Scale bar, 50 μm. **(B)** Quantification of positive staining in **(A)**, presented as percentage of total tissue area (*n* = 3), analyzed using ImageJ. **(C)** Representative IHC staining for complement component C3 in lung sections from WT and β3^−/−^ mice. Scale bar, 50 μm. **(D)** Quantification of positive staining in **(C)**, presented as percentage of total tissue area (*n* = 3), analyzed using ImageJ. **(E)** Representative immunofluorescence (IF) images showing the colocalization of IgG (green) and C3 (red) deposits in β3^−/−^ lungs. Nuclei were counterstained with DAPI (blue). The merged yellow signal indicates the formation of immune complexes (indicated by arrows). Scale bar, 50 μm. **(F, G)** Representative flow cytometry dot plots showing the expression of IgM **(F)** and IgG1 **(G)** on pulmonary B cells (all gated on live CD45^+^ CD19^+^ cells) from WT and β3^−/−^ mice. **(H)** Quantitative analysis of the percentages of IgM^+^ and IgG1^+^ B cells within the total B cell population (n = 3). **(I, J)** Representative flow cytometry dot plots showing the expression of the activation marker CD80 **(I)** and memory marker PD-L2 **(J)** on pulmonary B cells (gated on live CD45^+^ CD19^+^ cells). **(K)** Quantitative analysis of the percentages of CD80^+^ and PD-L2^+^ B cells within the total B cell population (n = 3). Data are presented as mean ± SD. Statistical significance was determined by two-tailed Student’s *t*-test (GraphPad Prism).

Guided by this multi-omics evidence pointing to the role of humoral immunity, we then investigated lung tissues for signs of B cell activation and effector function. Immunohistochemically stained for CD19 showed an increased number as well as clustering of B cells in inflamed lung parenchyma, indicative of local activation (**Figures 5A, B**). Since activated B cells drive antibody production, we then tested for downstream effector processes such as complement activation. The widespread deposition of complement factor C3 in lung tissue from β3^−/−^ mice (**Figures 5C, D**) was observed. Additionally, immunofluorescence showed a distinct localization of IgG together with C3 in the alveolar septae (**Figure 5E**), strongly arguing for *in situ* immune complex formation being a direct pathological consequence of increased humoral immune activity (**Figure 5E**).

To further validate the B cell landscape, we performed flow cytometric analysis on lung single-cell suspensions. Compared to WT controls, the B cell pool from β3^−/−^ mice displayed a significant decrease in the proportion of IgM^+^ B cells and an expansion of class-switched IgG1^+^ B cells (**Figures 5F–H**). Furthermore, both the costimulatory molecule CD80 and the memory marker PD-L2 were significantly upregulated in β3^−/−^ mice, indicating that a subset of β3^−/−^ B cells acquired a mature and activated phenotype (**Figures 5I–K**). Collectively, these findings demonstrate that β3^−/−^ pulmonary B cells are activated, with subsets successfully undergoing class-switch recombination and differentiating into memory phenotypes.

**Figure 6:**
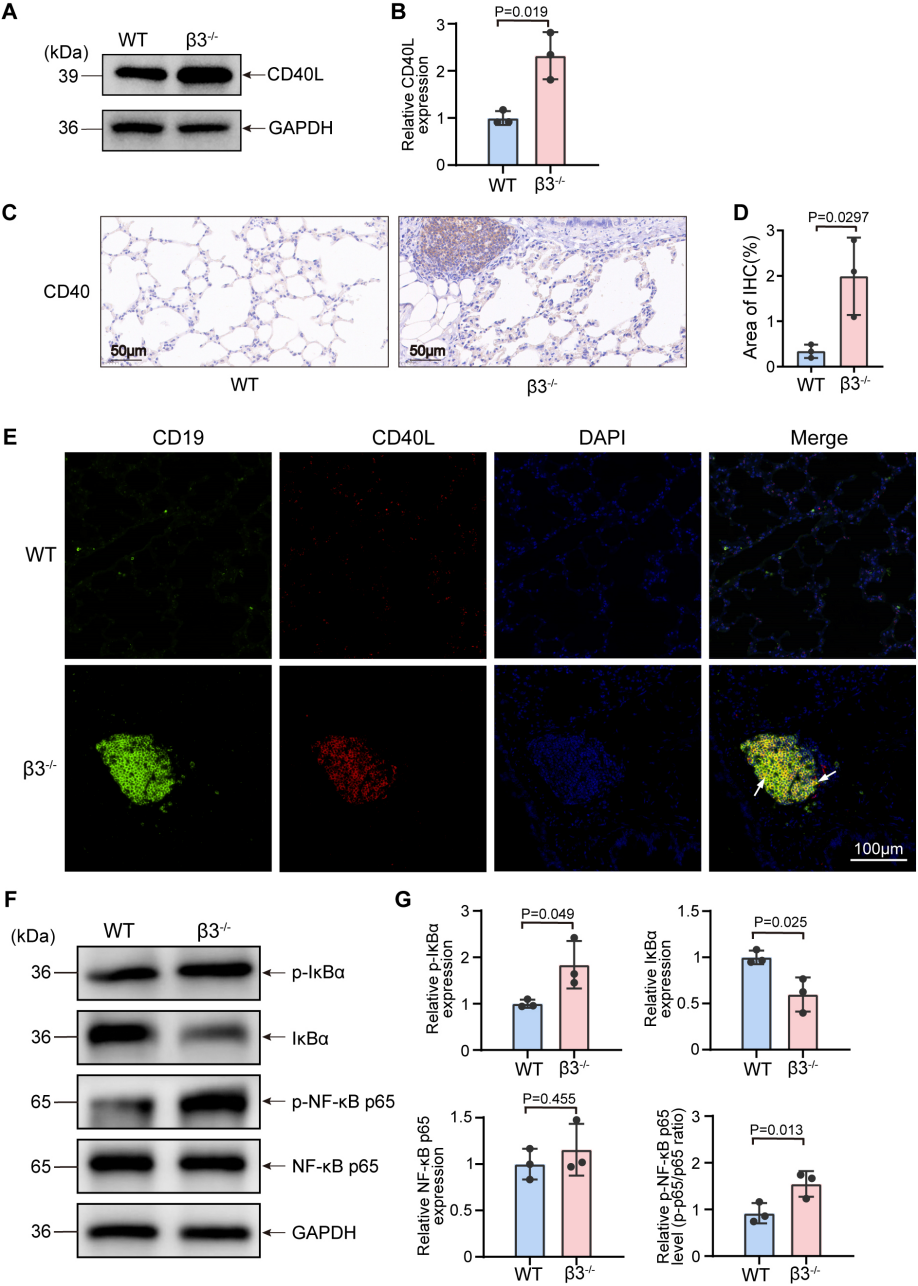
Hyperactivation of the CD40L-CD40 axis and downstream NF-κB signaling in β3^-/-^ lungs. **(A)** Western blot analysis of CD40L expression in whole lung lysates. GAPDH served as the loading control. **(B)** Quantification of CD40L protein levels (*n* = 3). **(C)** Representative IHC staining for the CD40 receptor in lung sections from WT and β3^−/−^ mice. Scale bar, 50 μm. **(D)** Quantification of positive staining in **(C)**, presented as percentage of total tissue area (*n* = 3), analyzed using ImageJ. **(E)** Representative immunofluorescence (IF) images showing the colocalization of CD19 (green) and CD40L (red) deposits in β3^−/−^ lungs. Nuclei were counterstained with DAPI (blue). The merged yellow signal indicates the formation of immune complexes (indicated by arrows). Scale bar, 100 μm. **(F)** Western blot analysis of the canonical NF-κB signaling pathway, assessing levels of phosphorylated IκBα (p-IκBα), total IκBα, phosphorylated NF-κB p65 (p-p65), and total NF-κB p65. **(G)** Quantification of the p-IκBα, IκBα, p-p65, and p65 protein levels (*n* = 3). Data are presented as mean ± SD. Statistical significance was determined by two-tailed Student’s *t*-test (GraphPad Prism).

Next, we tested the hypothesis derived from our protein–protein interaction analysis that the CD40-CD40L signaling axis was functionally enhanced in β3^−/−^ lungs. Western blot analysis demonstrated significant upregulation of CD40L protein expression in whole-lung lysates isolated from β3^−/−^ mice (**Figures 6A, B**). IHC showed increased CD40 expression that was primarily found in areas with infiltrating inflammatory cells (**Figures 6C, D**).The immunofluorescence co-localization data also suggested that there is often some co-localization of the CD40L with CD19^+^ B cell aggregates (**Figure 6E**), consistent with an explanation whereby the increased levels of CD40L are binding to the CD40 expressed by B-cells, thus enhancing the activation of B cells and promoting a humoral immune–mediated lung pathology.

### 3.6 Activation of the canonical NF-κB pathway downstream of the CD40-CD40L axis

CD40 ligation potently activates the canonical NF-κB signaling pathway (24, 25), a central regulator of inflammatory responses that was also highlighted in our proteomic KEGG analysis. We therefore examined the activation status of this pathway as a key convergent downstream mechanism.

The Western blot results showed that the expression level of p-IκBα was significantly increased and the expression level of total IκBα protein decreased in lung tissue of β3^−/−^ mice (**Figures 6F, G**). Such an increased degradation of IκBα favors NF-κB activation by allowing a translocation in nucleus of NF-κB complex itself. In agreement with this, we found an increased level of phospho-NF-κB p65 (phospho-p65) but not total p65 (**Figures 6F, G**). Taken together, our results indicate that there is significant stimulation of the classic NF-κB pathway and suggest an intracellular pathway by which increased CD40 signaling may result in continued production of inflammatory cytokines and chronic lung inflammation.

### 3.7 Downregulation of *ITGB3* is correlated with B cell hyperactivation in human malignancies and autoimmune diseases

To establish the translational relevance of the mechanistic pathway identified in our murine models, we conducted a comprehensive bioinformatic analysis of publicly available transcriptomic datasets spanning diverse human malignancies and systemic autoimmune diseases.

**Figure 7:**
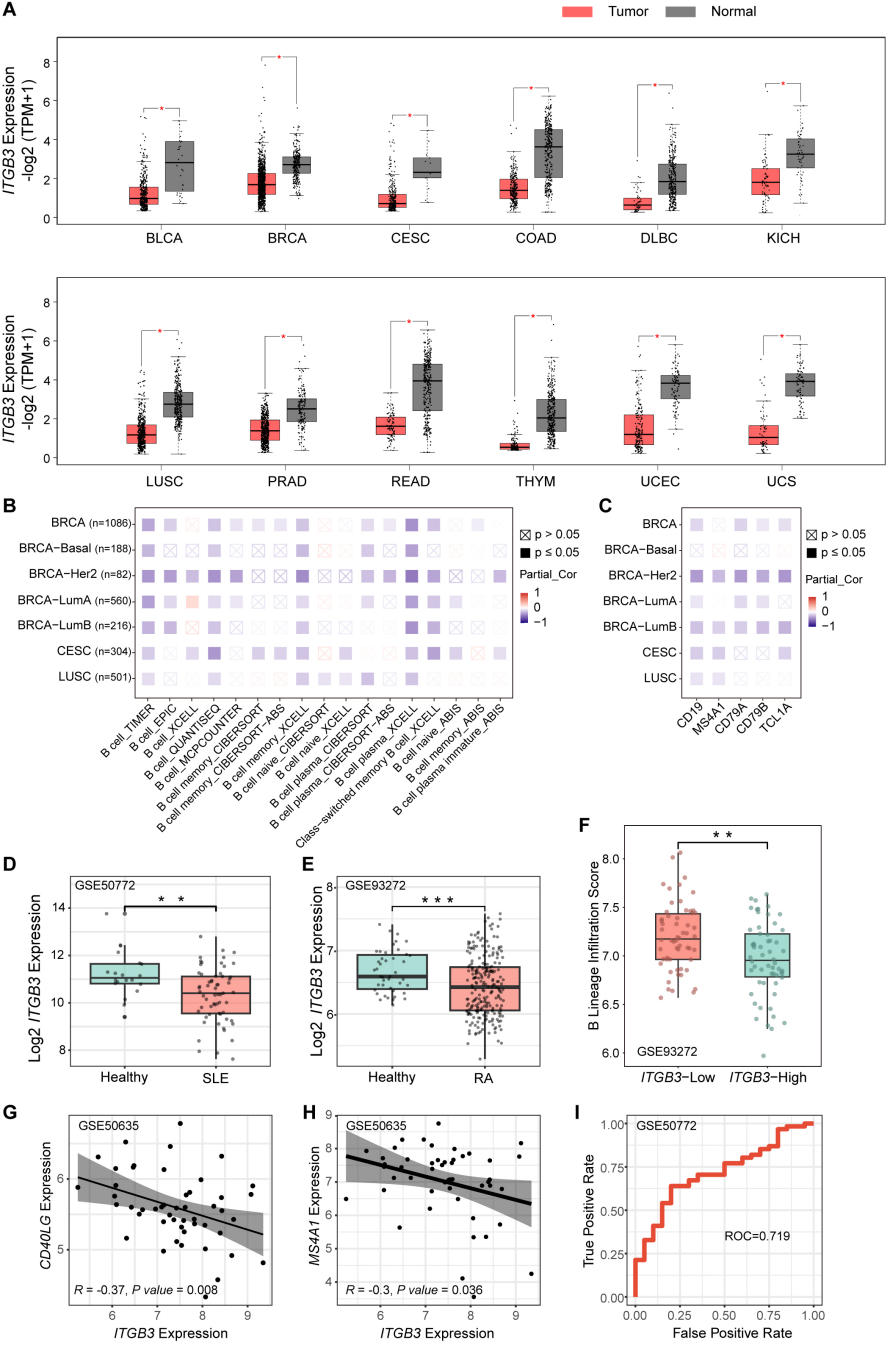
Downregulation of *ITGB3* is associated with B cell hyperactivation in human malignancies and autoimmune diseases. **(A)** Pan-cancer analysis of *ITGB3* expression in tumor tissues (red) versus adjacent normal tissues (gray). Data were sourced from the TCGA and GTEx databases. BLCA, Bladder Urothelial Carcinoma; BRCA, Breast Invasive Carcinoma; CESC, Cervical Squamous Cell Carcinoma and Endocervical Adenocarcinoma; COAD, Colon Adenocarcinoma; DLBC, Diffuse Large B-cell Lymphoma; KICH, Kidney Chromophobe; LUSC, Lung Squamous Cell Carcinoma; PRAD, Prostate Adenocarcinoma; READ, Rectum Adenocarcinoma; THYM, Thymoma; UCEC, Uterine Corpus Endometrial Carcinoma; UCS, Uterine Carcinosarcoma. **(B)** Correlation heatmap between *ITGB3* expression and levels of B cell immune infiltration in BRCA, CESC, and LUSC. Purple indicates a negative correlation; red indicates a positive correlation. Analyses were performed using TIMER3.0 with adjustment for tumor purity. **(C)** Correlation analysis between *ITGB3* expression and specific B cell lineage markers (*CD19*, *MS4A1*, *CD79A*, *CD79B*, and *TCL1A*) across the indicated cancer types. Data were analyzed using the TIMER3.0 database. **(D, E)** Validation of *ITGB3* downregulation in autoimmune diseases using GEO datasets. Box plots compare *ITGB3* mRNA levels between healthy controls (teal) and patients (salmon) in the GSE50772 SLE cohort **(D)** and the GSE93272 RA cohort **(E)**. **(F)** Validation of B cell enrichment in *ITGB3* deficient patients within the RA cohort (GSE93272). Patients were stratified into *ITGB3*-high and *ITGB3*-low groups. Boxplots depict the B cell lineage infiltration scores inferred from transcriptomic data. Statistical significance was determined by Wilcoxon rank-sum test. **(G, H)** Scatter plots demonstrate significant negative correlations between *ITGB3* expression and B cell activation markers **(G)** *CD40LG* and **(H)** *MS4A1* in the GSE50635 SLE cohort. Correlation coefficient R and P value were derived from Spearman analysis. **(I)** Receiver Operating Characteristic (ROC) curve assessing the diagnostic performance of ITGB3 expression in discriminating SLE patients from healthy controls (GSE50772). The Area Under the Curve is 0.719, indicative of fair diagnostic accuracy.

Pan-cancer analysis revealed a broad reduction in *ITGB3* expression, which encodes integrin β3, across multiple tumor types compared with corresponding normal tissues, suggesting that *ITGB3* downregulation commonly accompanies disrupted tissue homeostasis (**Figures 7A**; **Supplementary Figure 4**). Notably, in several malignancies including breast invasive carcinoma (BRCA), cervical squamous cell carcinoma and endocervical adenocarcinoma (CESC), and lung squamous cell carcinoma (LUSC), *ITGB3* expression showed a significant negative correlation with the extent of B cell infiltration (**Figure 7B**). Within these tumor microenvironments, *ITGB3* levels also inversely correlated with the expression of canonical B cell markers, including *CD19*, *MS4A1*, *CD79A*, *CD79B*, and *TCL1A* (**Figure 7C**). These associations indicate that reduced *ITGB3* expression may be linked to heightened B cell activation. Beyond malignancies, we extended our analysis to autoimmune diseases that share key pathological features with the phenotype observed in β3^−/−^ mice. We analyzed transcriptomic datasets from the GEO encompassing SLE and RA. Consistent with the pan-cancer findings, *ITGB3* expression was significantly reduced in peripheral blood mononuclear cells and whole-blood samples from patients with SLE and RA compared with healthy controls (**Figures 7D, E**).

To further define the immunoregulatory role of *ITGB3* in disease, we focused on the RA cohort. To account for interpatient heterogeneity, we stratified RA samples into *ITGB3*-high and *ITGB3*-low groups based on expression levels. Immune cell deconvolution analysis showed that the *ITGB3*-low group displayed significantly higher B cell infiltration scores than the *ITGB3*-high group (**Figure 7F**). Correlation analysis further revealed a strong inverse association between *ITGB3* expression and key markers of B cell activation. Specifically, *ITGB3* levels negatively correlated with *CD40LG* expression and with genes involved in B cell receptor signaling, including *MS4A1* (**Figures 7G, H**). These findings align with our mechanistic data, indicating that physiological β3 expression restrains CD40–CD40L–dependent B cell activation.

We next assessed the clinical relevance of *ITGB3* as a biomarker. ROC curve analysis demonstrated that *ITGB3* expression levels possessed potential diagnostic sensitivity and specificity for distinguishing SLE patients from healthy controls, supporting its diagnostic potential (**Figure 7I**). Together, these clinical observations reinforce the concept that *ITGB3* acts as a threshold regulator, and that its deficiency is a pathologically relevant feature correlated with B cell hyperactivation and disease progression in human autoimmune conditions.

## 4 Discussion

**Figure 8:**
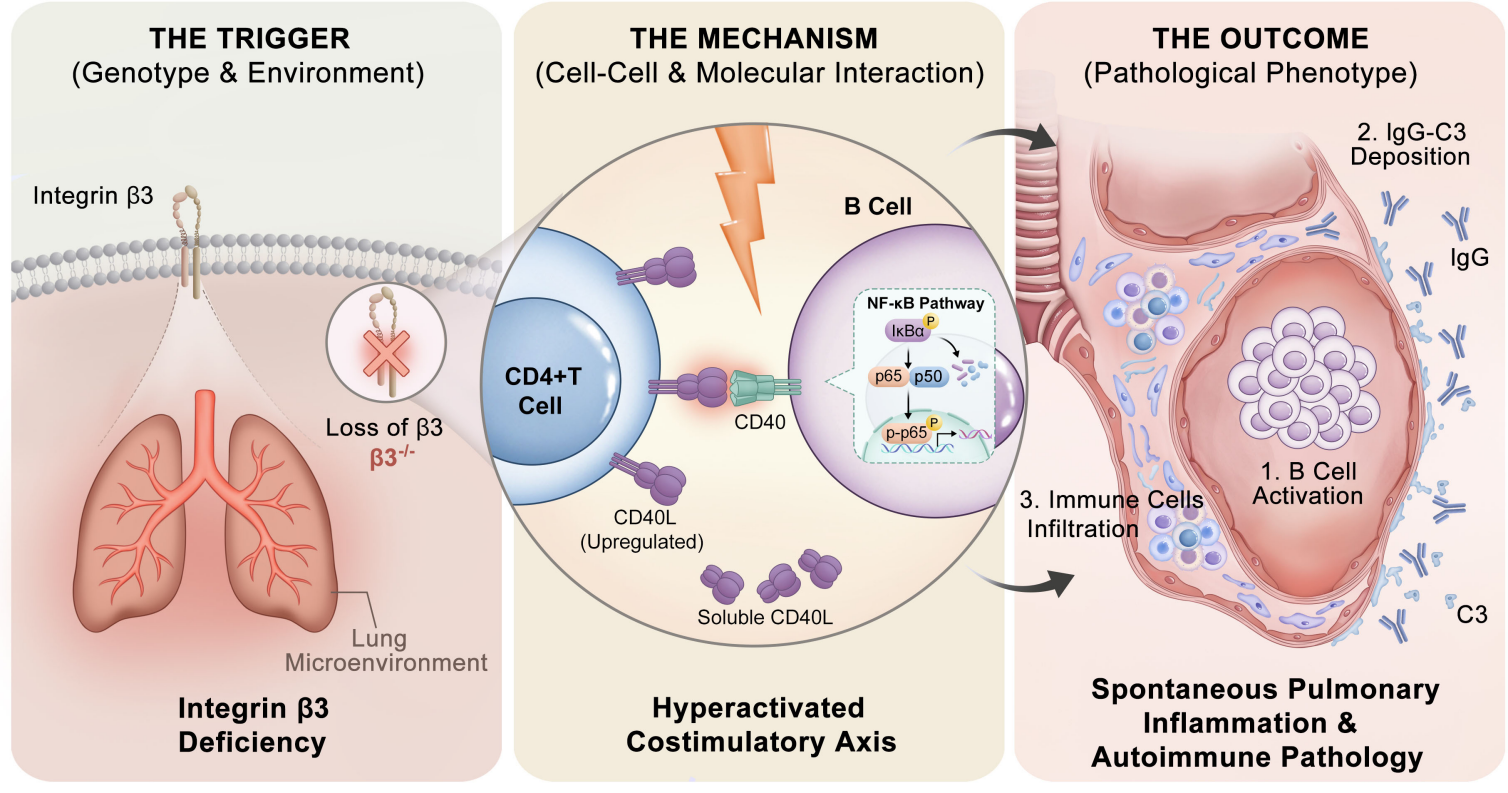
Working model: Integrin β3 deficiency promotes B cell-mediated pulmonary inflammation via the CD40-CD40L axis.

Maintenance of pulmonary immune homeostasis necessitates tight suppression of inappropriate immune responses to innocuous environmental antigens. While integrins are best known for their roles in cell adhesion and migration (6), their role in maintaining the immunological tolerance state within quiescent tissue is unclear. Here, we reveal that integrin β3 acts as an important physiological checkpoint for B cell activation during spontaneous lung inflammation. We show that the autoimmune disease caused by the genetic deletion of β3 is characterized by prominent extrafollicular B cell infiltration and localized IC deposition. Using complementary high-throughput multi-omics approaches, coupled to pathological confirmation, we associate this pathology with a loss of regulation in the CD40–CD40L costimulatory axis (**Figure 8**). Collectively, our results transform the role of integrin β3 away from that of a mere structural protein toward that of an important modulator of humoral homeostasis in the lung niche.

Our data highlight a previously unappreciated role for integrin β3 in restraining B cell activation evidenced through the inflammatory phenotype of β3^−/−^ mice. The β3^−/−^ mouse model was initially developed by Hodivala-Dilke et al. to characterize its defects in platelet aggregation and osteoclast function (26). Subsequent studies recognized that these animals are predisposed to lung inflammation; however, this has mostly been described as a response to metabolic stress (e.g., high-fat diet) and attributable to macrophage dysfunction or increased CD36 signaling on smooth muscle cells (9, 27). In distinct contrast, our results show that loss of β3 promotes ordered clustering of CD19^+^ B cells and *in situ* immune complex formation in the lung. This spatial organization resembles inducible bronchus-associated lymphoid tissue (iBALT), a defining feature of chronic autoimmune lung diseases such as RA-ILD (28, 29). These findings place integrin β3 in a position to act as an upstream regulator limiting B-cell activation in the pulmonary environment.

Our unbiased transcriptomic and proteomic analyses consistently identified CD40L as a central driver of the observed immune dysregulation. Under physiological conditions, CD40L expression is transient and tightly controlled to prevent excessive immune activation (30). In contrast, β3^−/−^ lungs exhibited sustained CD40L upregulation, indicating a critical breakdown in this regulatory system. How loss of β3 mechanistically leads to persistent CD40L elevation therefore represents a key question arising from our findings.

The precise molecular hierarchy linking β3 loss to sustained CD40L upregulation probably involves a combination of indirect microenvironmental defects and direct structural constraints. Our transcriptomic data (**Figure 2G**) revealed an upregulation of *Cd40lg* mRNA, suggesting that the elevated CD40L protein levels are driven by transcriptional upregulation. αvβ3 is the canonical receptor for the activation of latent TGF-β (31–33) and efferocytosis (clearance of apoptotic cells) (34). In β3^−/−^ lungs, the local depletion of bioactive TGF-β, a potent repressor of T cell activation, would lead to the unleashing of *Cd40lg* transcription (35, 36). Furthermore, defective clearance of apoptotic debris would lead to secondary necrosis and the release of autoantigens.

Additionally, integrin β3 may act as a negative regulator of CD40L bioavailability for a collaborative mechanism. Soluble CD40L contains an RGD motif, enabling high-affinity binding to β3-containing integrins (αvβ3 on immune cells and αIIbβ3 on platelets) (37, 38). Under homeostatic conditions, β3 expression likely sequesters CD40L and limits its availability to engage the CD40 receptor. In the absence of this constraint, CD40L can interact freely with CD40, explaining the hyperactivation of the downstream NF-κB cascade. These mechanisms establish β3 as a multidimensional regulator of immune homeostasis.

Although the most pronounced pathology manifests in the lung, our multi-omics examination of splenic tissue shows that β3 deficiency causes an overall immune disturbance (**Figure 4**), and the primed state of splenic lymphocytes indicates that lack of β3 reduces the overall threshold required to activate the immune system. To explain why pathology is observed mainly in the lung, we suggest a “Two-Hit” model. The first hit is the genetic loss of β3 establishing a systemic predisposition for autoimmunity. It is precisely due to continuous environmental stimulation that the second hit occurs specifically in the lung. In this uniquely susceptible environment, lacking the regulation normally afforded by β3, is a persistent antigen stimulation leading to an inflammatory reaction. It shows that while the defect is systemic, the triggering of the clinical onset depends on local environmental factors.

The constellation of pathological features observed in β3^−/−^ lungs including organized clustering of CD19^+^ B cells and deposition of IgG–C3 immune complexes closely mirrors the mechanisms underlying antibody-mediated autoimmune diseases such as systemic lupus erythematosus and rheumatoid arthritis (39, 40). Inflammatory infiltrates of the β3^−/−^ lungs are well organized as inducible bronchus associated lymphoid tissue (iBALT) like structures instead of diffuse ones. Thus, these results reveal that β3 signaling acts as an important barrier to *de novo* lymphoid aggregate formation within the lung. As such, the β3^−/−^ mouse is an important preclinical model to dissect the mechanisms by which integrin dysfunction promotes B cell–dependent pulmonary autoimmunity.

To strengthen the translational relevance of this regulatory axis, we analyzed publicly available transcriptomic datasets from TCGA and GEO spanning a broad range of human lung pathologies. Across autoimmune diseases and multiple cancer types, *ITGB3* expression was consistently reduced, suggesting that loss of this integrin represents a common feature of disrupted tissue homeostasis. In malignancies, previous studies have largely emphasized β3 integrin functions in cell adhesion, migration, and angiogenesis (41, 42). In contrast, our pan-cancer analysis revealed a negative association between *ITGB3* expression and B cell infiltration and activation. This finding establishes a previously unrecognized link between integrin signaling and the tumor immune microenvironment and expands the functional scope of β3 to include regulation of adaptive immunity. Importantly, *ITGB3* expression also showed a robust inverse correlation with B cell–driven inflammatory signatures in autoimmune diseases, particularly those associated with the CD40–CD40L pathway. These human data closely mirror our murine observations and support a conserved role for β3 as a physiological brake on B cell activation. From a clinical perspective, *ITGB3* demonstrates potential utility as a prognostic and stratification biomarker. Low *ITGB3* expression displayed high diagnostic sensitivity in our analyses, suggesting that *ITGB3* may help identify patient subsets characterized by B cell hyperactivation. Therefore, clinical investigations measuring *ITGB3* expression levels could serve as a potential diagnostic biomarker. Identifying patients with this specific biomarker signature would help clinicians recognize distinct inflammatory conditions driven by B cell hyperactivation and accurately guide the use of targeted therapies.

We recognize several limitations of this study. First and foremost, the use of a constitutive β3 knockout model precludes definitive identification of the initiating cellular lineage. While our data highlight B cells as the primary effector cells driving the inflammatory phenotype, we cannot exclude the possibility that B cell hyperactivation arises secondarily to alterations in the lung microenvironment or defects in other immune and structural cells. For instance, β3 is critical for macrophage-mediated efferocytosis (43). Defective clearance in β3^−/−^ lungs could lead to the accumulation of secondary necrotic debris and autoantigens, thereby providing a chronic stimulus for B cell activation. Similarly, β3 deficiency in endothelial cells may compromise vascular integrity or alter lymphocyte trafficking (44), while defects in platelets could disrupt their immunomodulatory interaction with adaptive immune cells via CD40L release (45). Thus, the observed pathology likely results from a compound effect. Future studies using lineage-specific knockout strategies, such as CD19-Cre for B cells, PF4-Cre for platelets, or Tek-Cre for endothelial cells, will be necessary to dissect the precise contribution of each compartment.

Second, while our transcriptomic and histological data suggest that the B cell compartment is functionally biased towards antibody-secreting plasma cells, we acknowledge that the exact frequencies of specific B cell subsets were not precisely quantified. Consequently, future research could employ single-cell RNA sequencing or high-dimensional spectral flow cytometry to enable high-resolution mapping of B cell developmental trajectories. Next, this study is limited by the absence of *in vivo* functional blockade data to confirm the therapeutic reversibility of the phenotype. To address this, confirmatory experiments using blocking CD40L antibodies are currently underway to validate the efficacy of targeting this axis. Finally, our clinical observations rely on transcriptomic correlations derived from cross-sectional datasets. While the inverse association between *ITGB3* expression and B cell activation signatures is robust, longitudinal analyses and prospective clinical studies will be required to establish causality and validate *ITGB3* as a predictive biomarker in human autoimmune disease.

We identify integrin β3 as a critical threshold regulator of B cell activation in pulmonary adaptive immunity. Loss of β3 disrupts B cell tolerance by amplifying the CD40–CD40L–NF-κB signaling axis, leading to spontaneous inflammatory pathology. The strong association between reduced *ITGB3* expression and B cell hyperactivation in patients with autoimmune disease underscores the translational relevance of our findings. Together, our results position integrin β3 as both a candidate biomarker for patient stratification and a promising therapeutic target for antibody-mediated autoimmune lung diseases.

## Data Availability

The datasets presented in this study can be found in online repositories. The names of the repository/repositories and accession number(s) can be found below: https://www.ncbi.nlm.nih.gov/geo/, GSE317568.
