# Supplementary figures and images for: Integrin β3 deficiency unleashes spontaneous pulmonary inflammation by promoting B cell hyperactivation via the CD40-CD40L axis
Source: Front Immunol. 2026 Mar 24;17:1796926. doi: 10.3389/fimmu.2026.1796926 (PMC13055533; doi:10.3389/fimmu.2026.1796926)

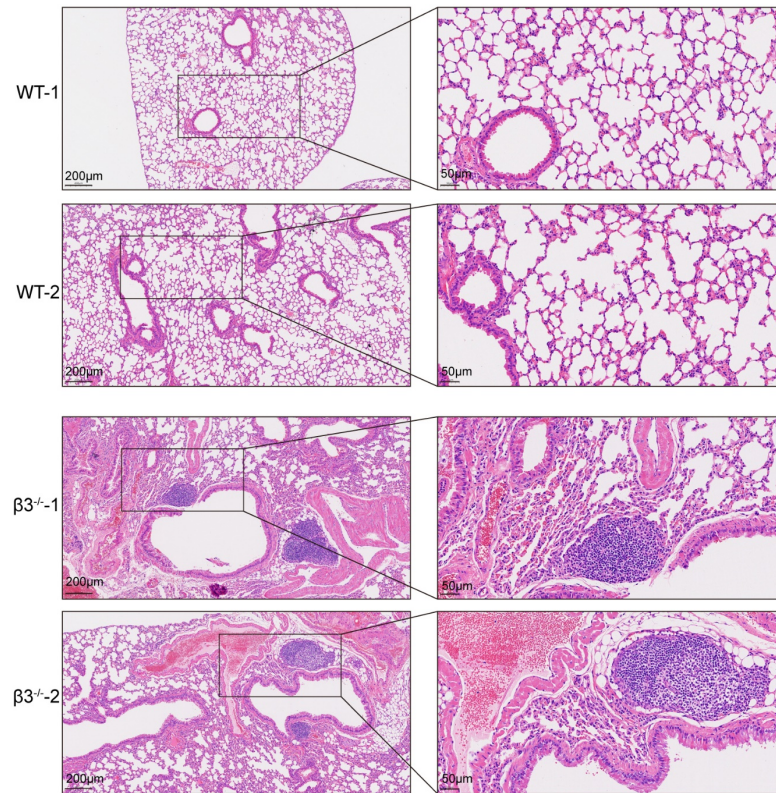

**Supplementary Figure 1. Hematoxylin and eosin (HE) staining of lung sections from WT and  $\beta 3^{-/-}$  mice.**

Supplement: Supplementary file 1 [file Image1.pdf]

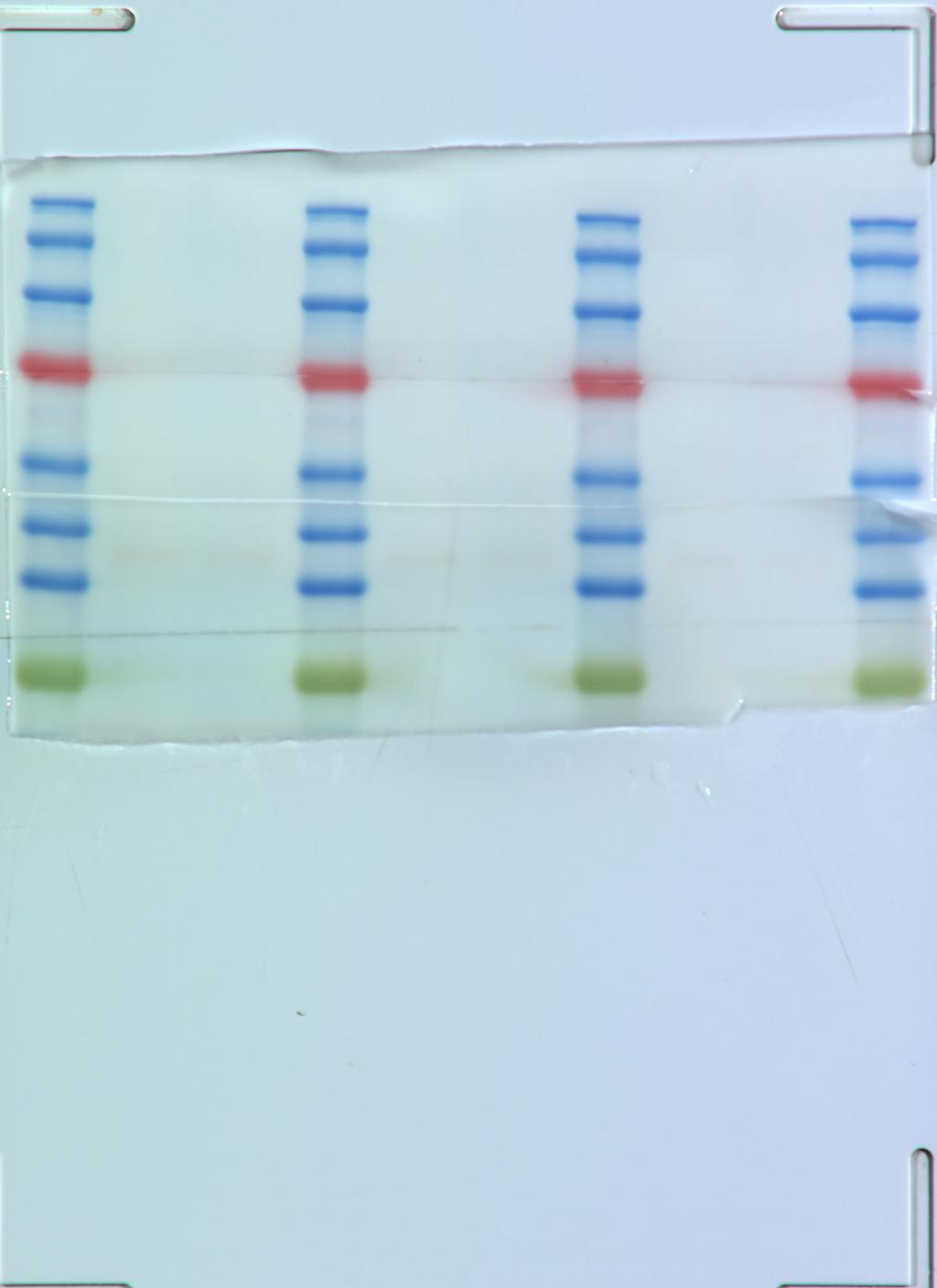

Supplement: Supplementary file 5 [file DataSheet1.zip › Raw data-WB images/Figure 1A/Figure 1A-All.jpg]

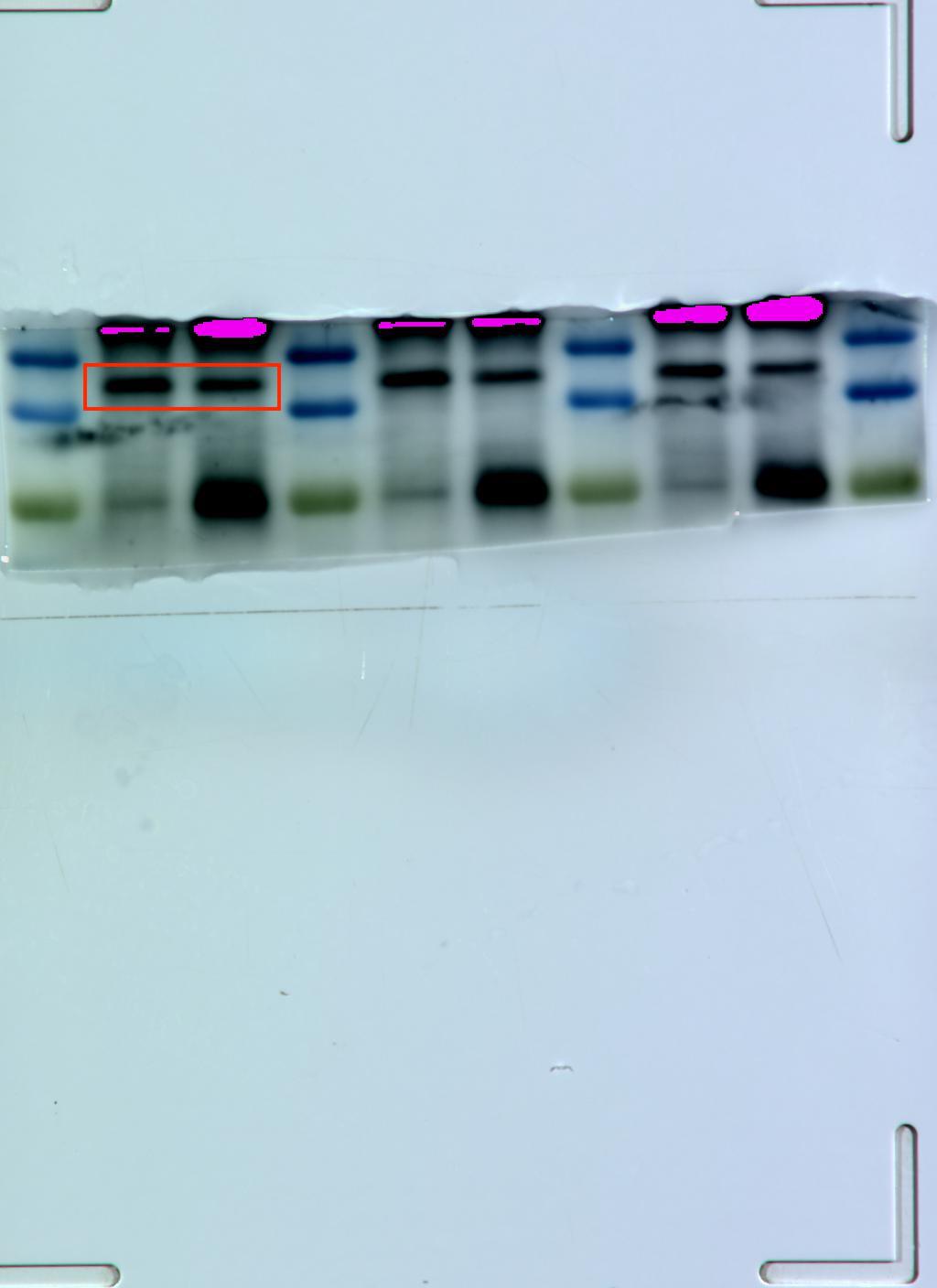

Supplement: Supplementary file 5 [file DataSheet1.zip › Raw data-WB images/Figure 1A/Figure 1A-GAPDH- region.jpg]

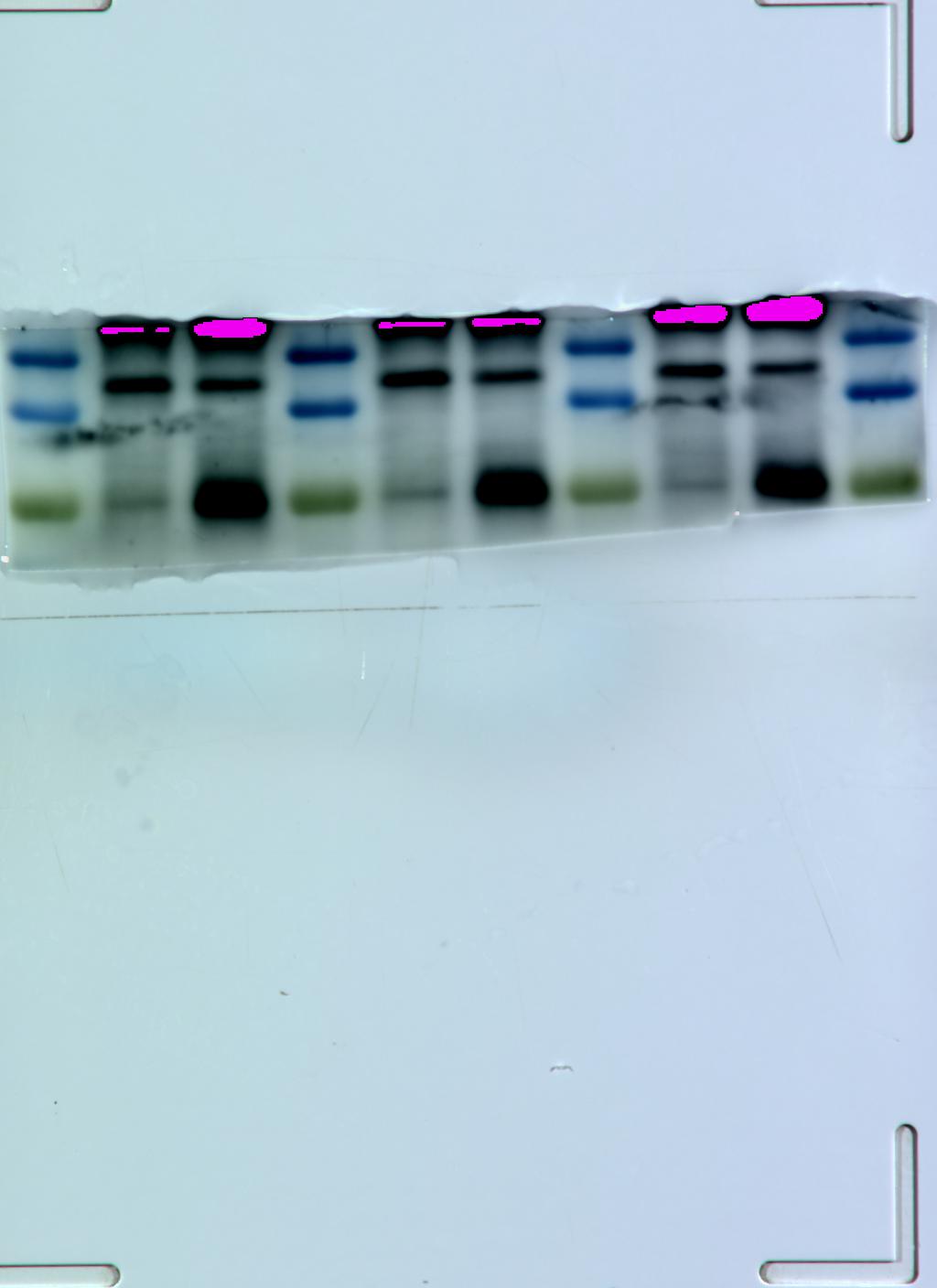

Supplement: Supplementary file 5 [file DataSheet1.zip › Raw data-WB images/Figure 1A/Figure 1A-GAPDH.jpg]

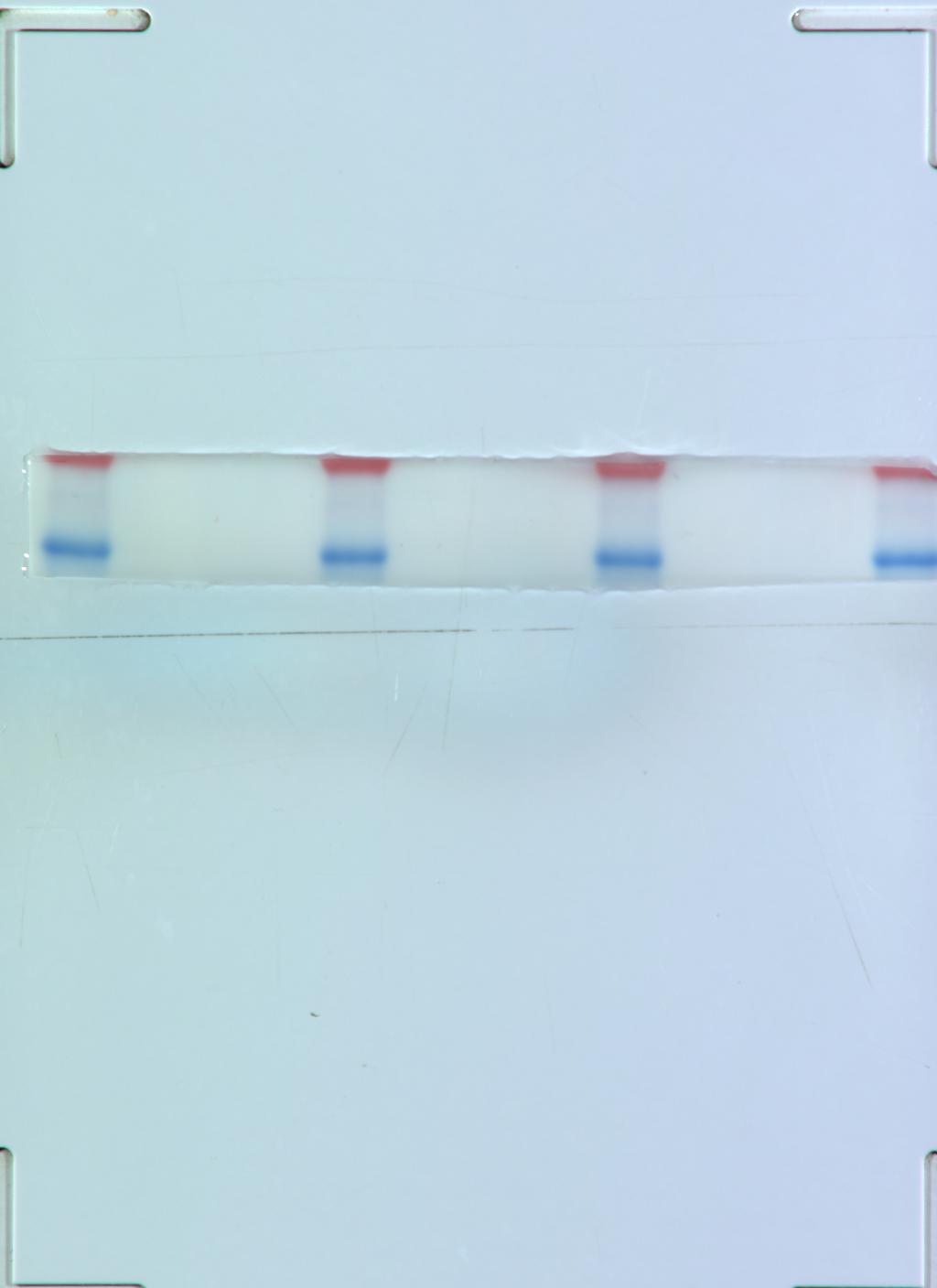

Supplement: Supplementary file 5 [file DataSheet1.zip › Raw data-WB images/Figure 1A/Figure 1A-other.jpg]

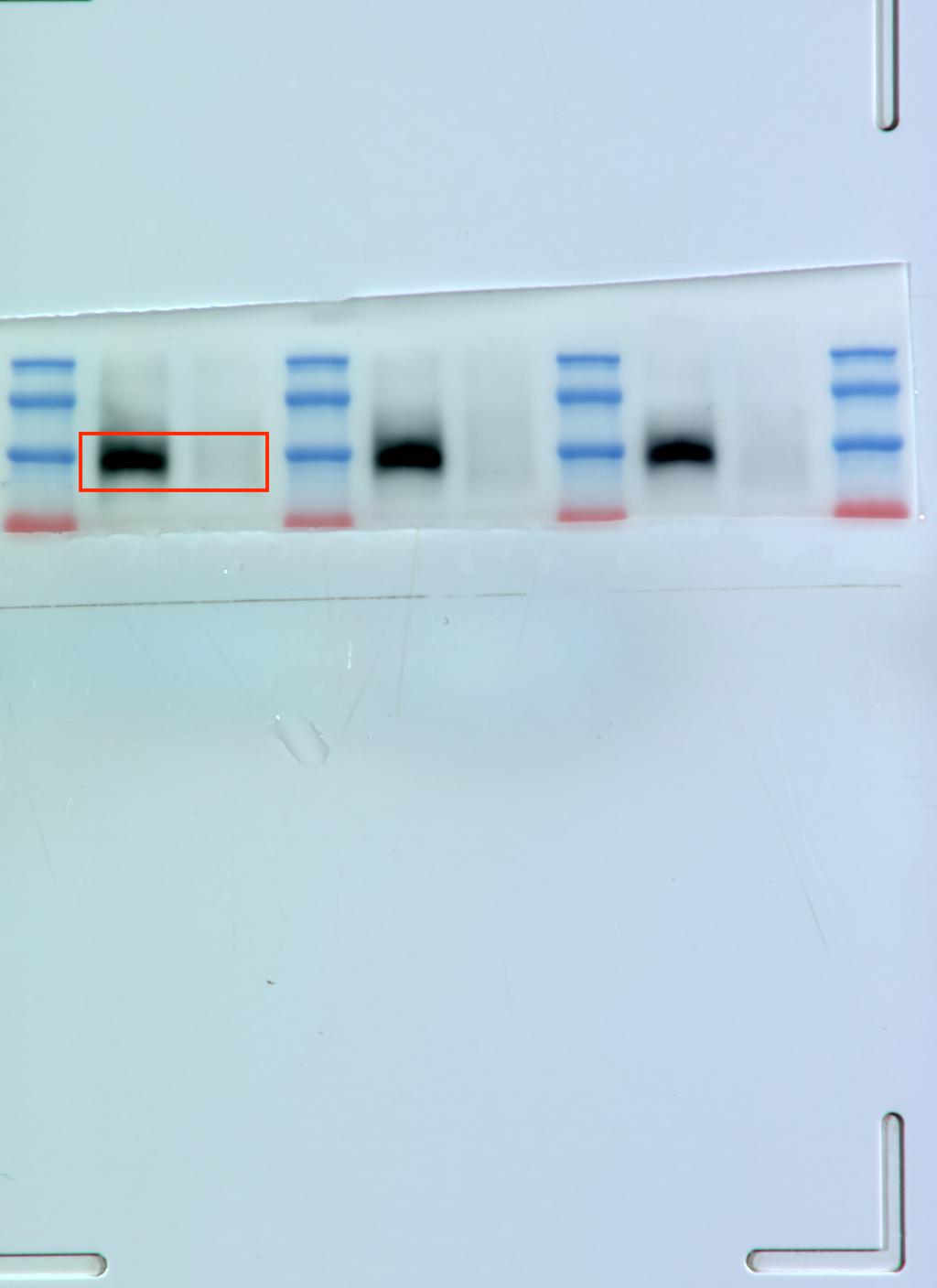

Supplement: Supplementary file 5 [file DataSheet1.zip › Raw data-WB images/Figure 1A/Figure 1A-β3- region.jpg]

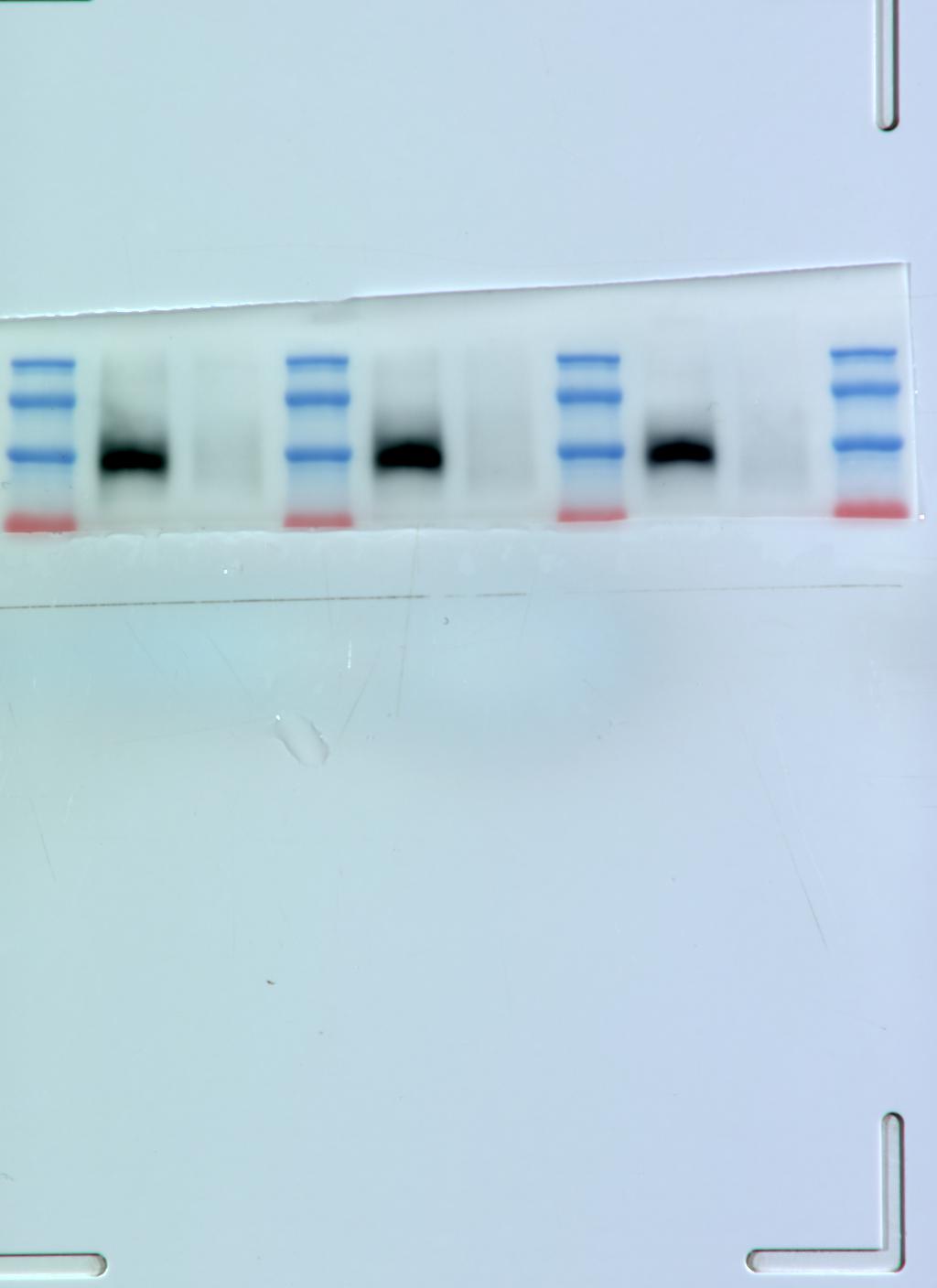

Supplement: Supplementary file 5 [file DataSheet1.zip › Raw data-WB images/Figure 1A/Figure 1A-β3.jpg]

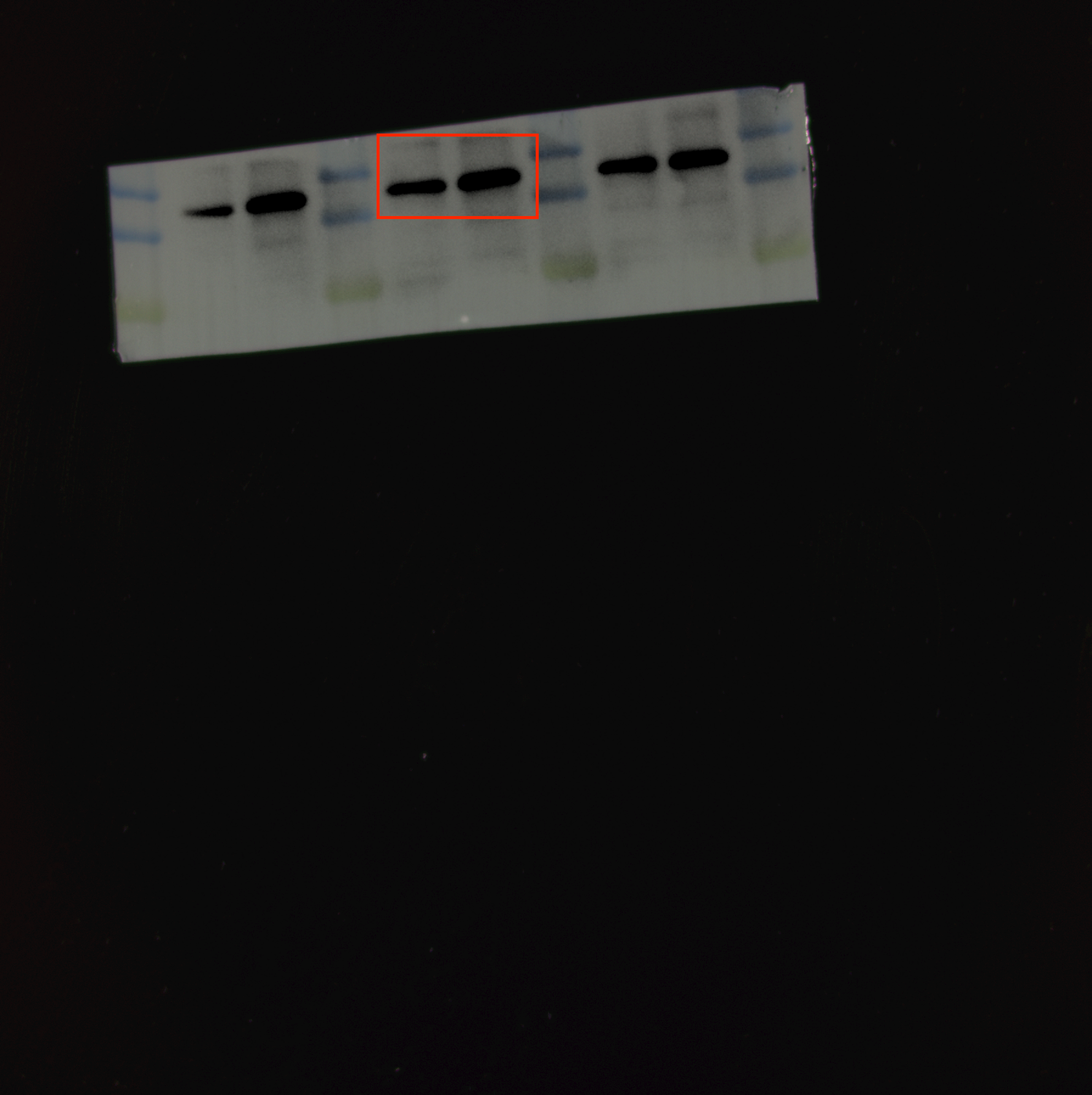

Supplement: Supplementary file 5 [file DataSheet1.zip › Raw data-WB images/Figure 6A/Figure 6A-CD40L- region.tiff]

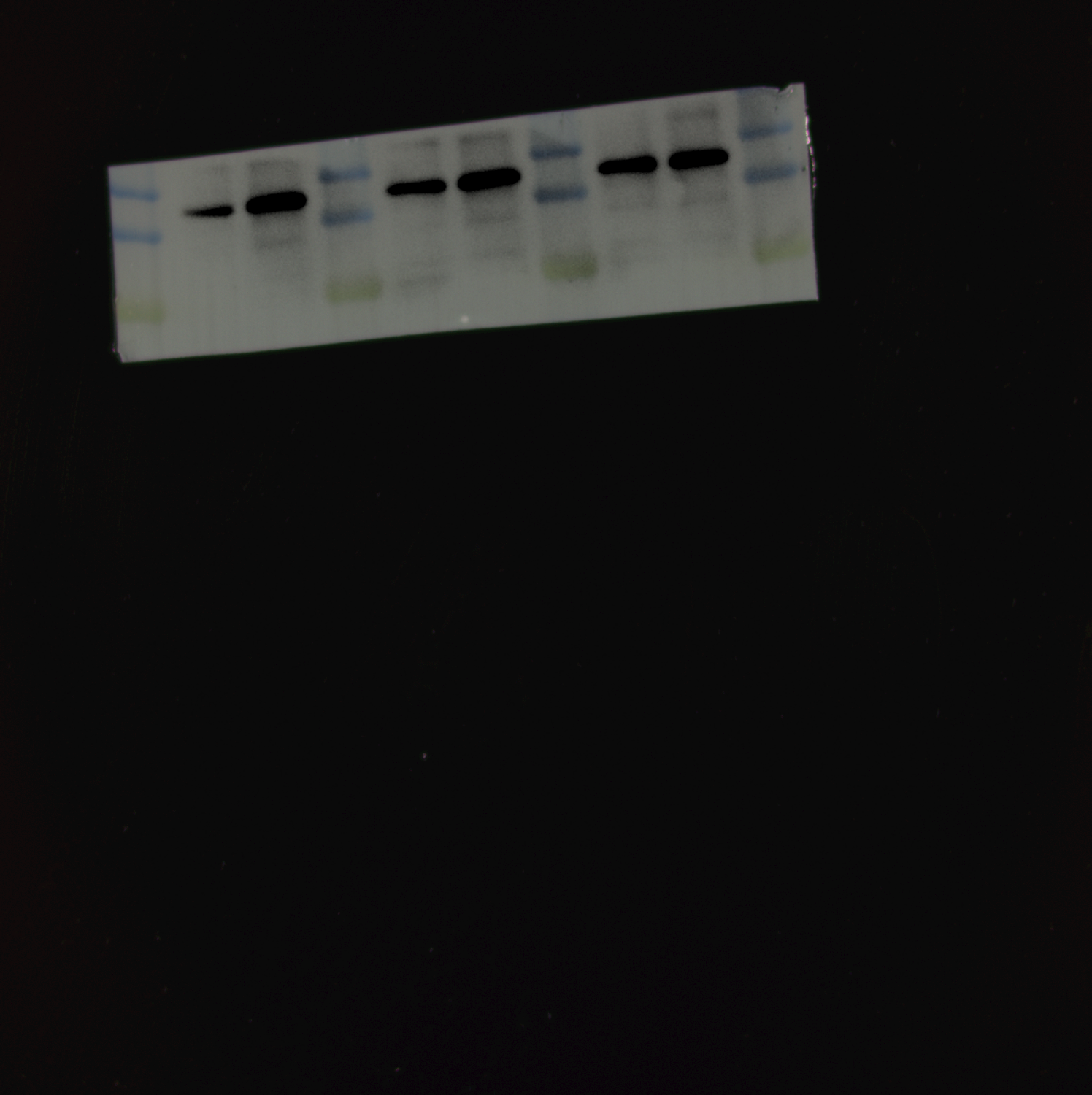

Supplement: Supplementary file 5 [file DataSheet1.zip › Raw data-WB images/Figure 6A/Figure 6A-CD40L.tiff]

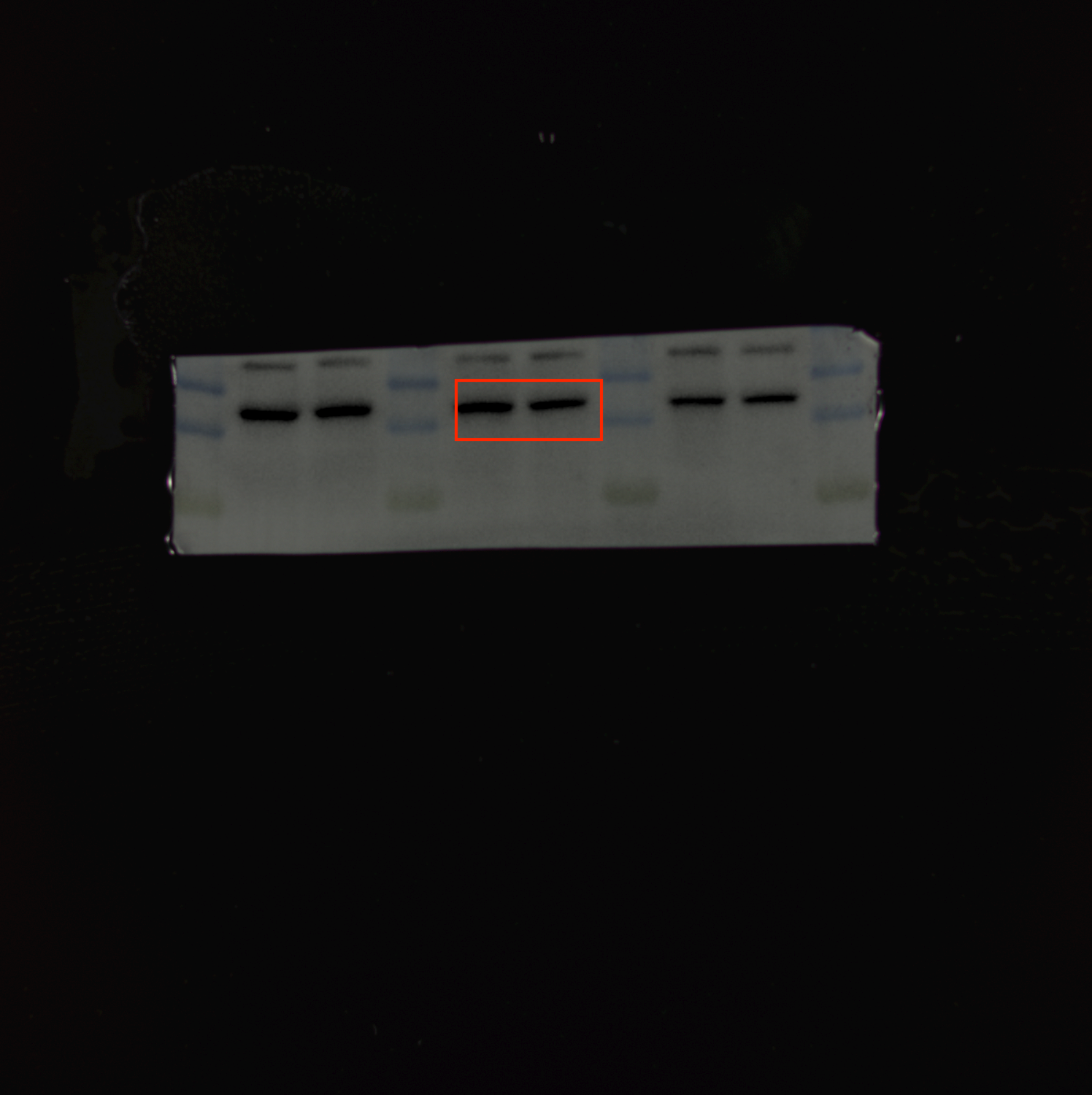

Supplement: Supplementary file 5 [file DataSheet1.zip › Raw data-WB images/Figure 6A/Figure 6A-GAPDH- region.tiff]

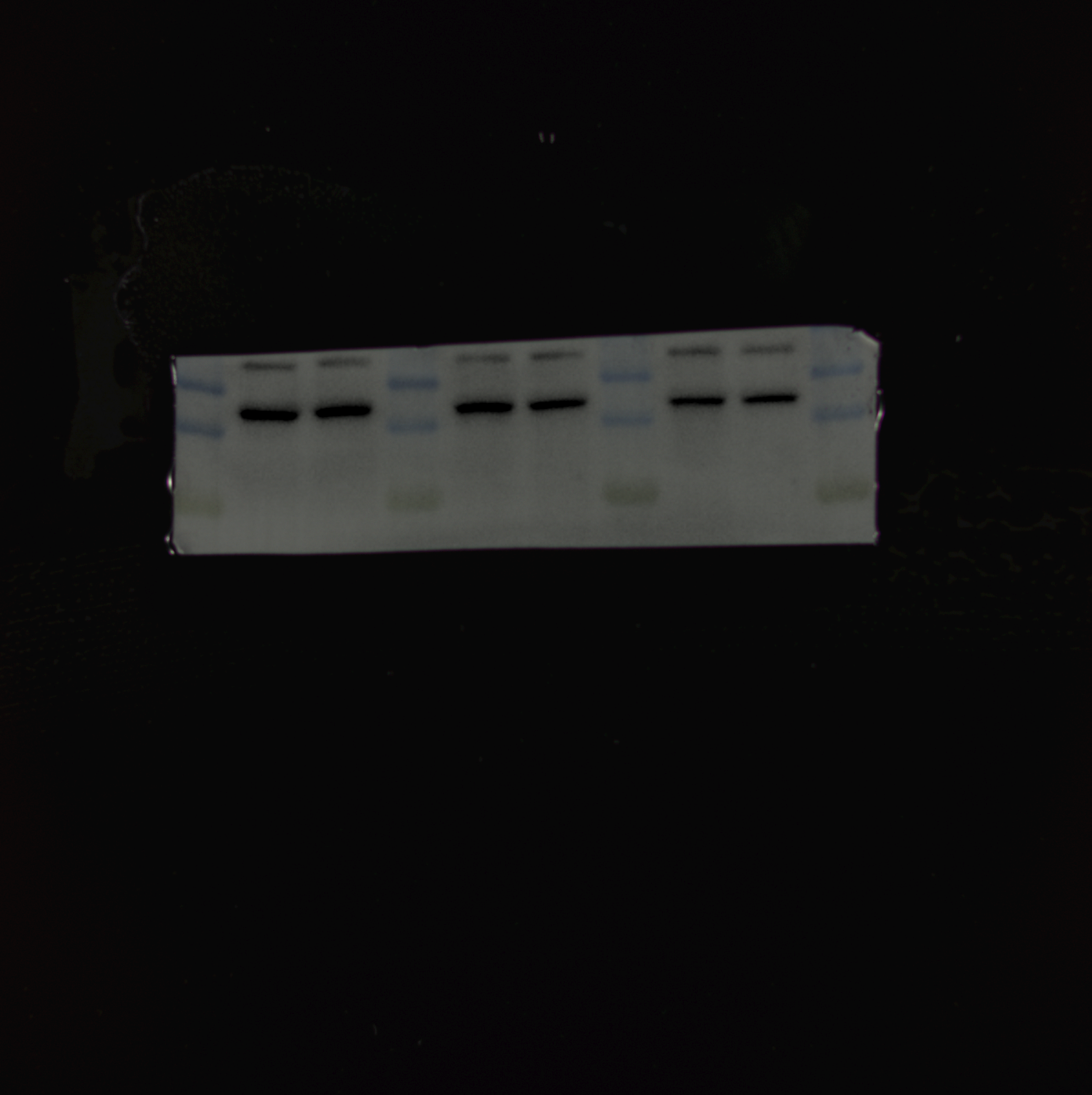

Supplement: Supplementary file 5 [file DataSheet1.zip › Raw data-WB images/Figure 6A/Figure 6A-GAPDH.tiff]

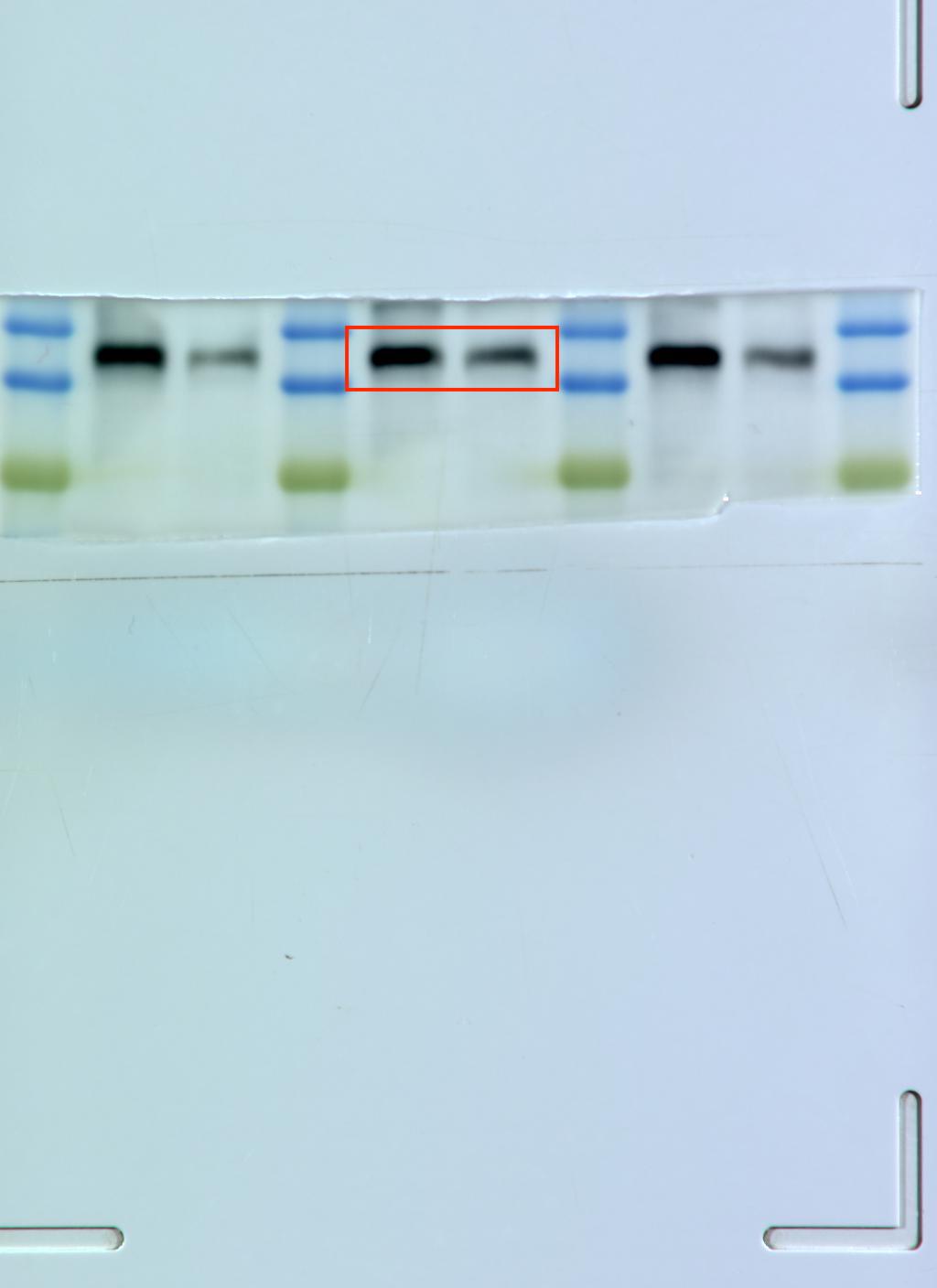

Supplement: Supplementary file 5 [file DataSheet1.zip › Raw data-WB images/Figure 6F/Figure 6F-IκBα/Figure 6F-IκBα- region.jpg]

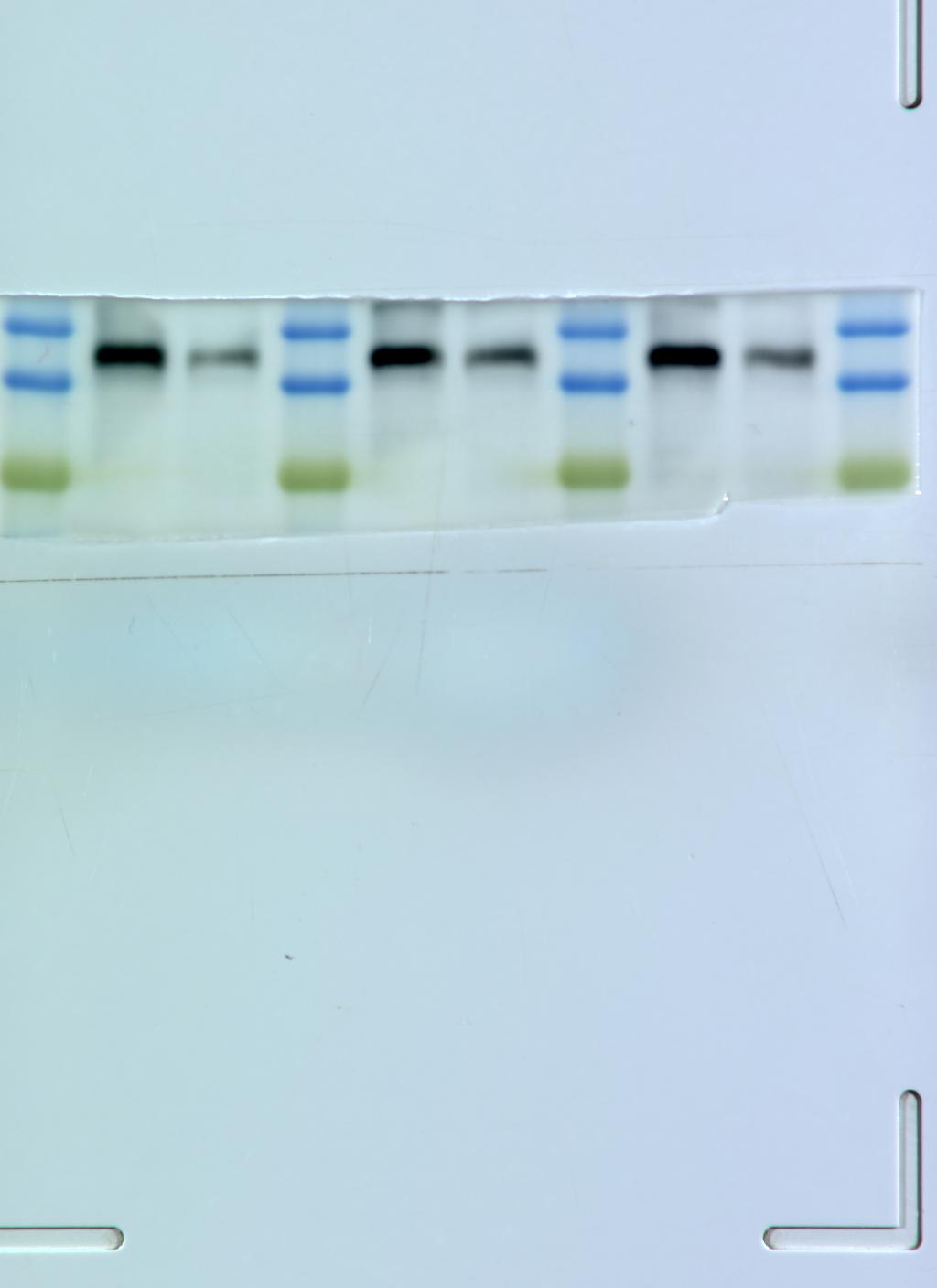

Supplement: Supplementary file 5 [file DataSheet1.zip › Raw data-WB images/Figure 6F/Figure 6F-IκBα/Figure 6F-IκBα.jpg]

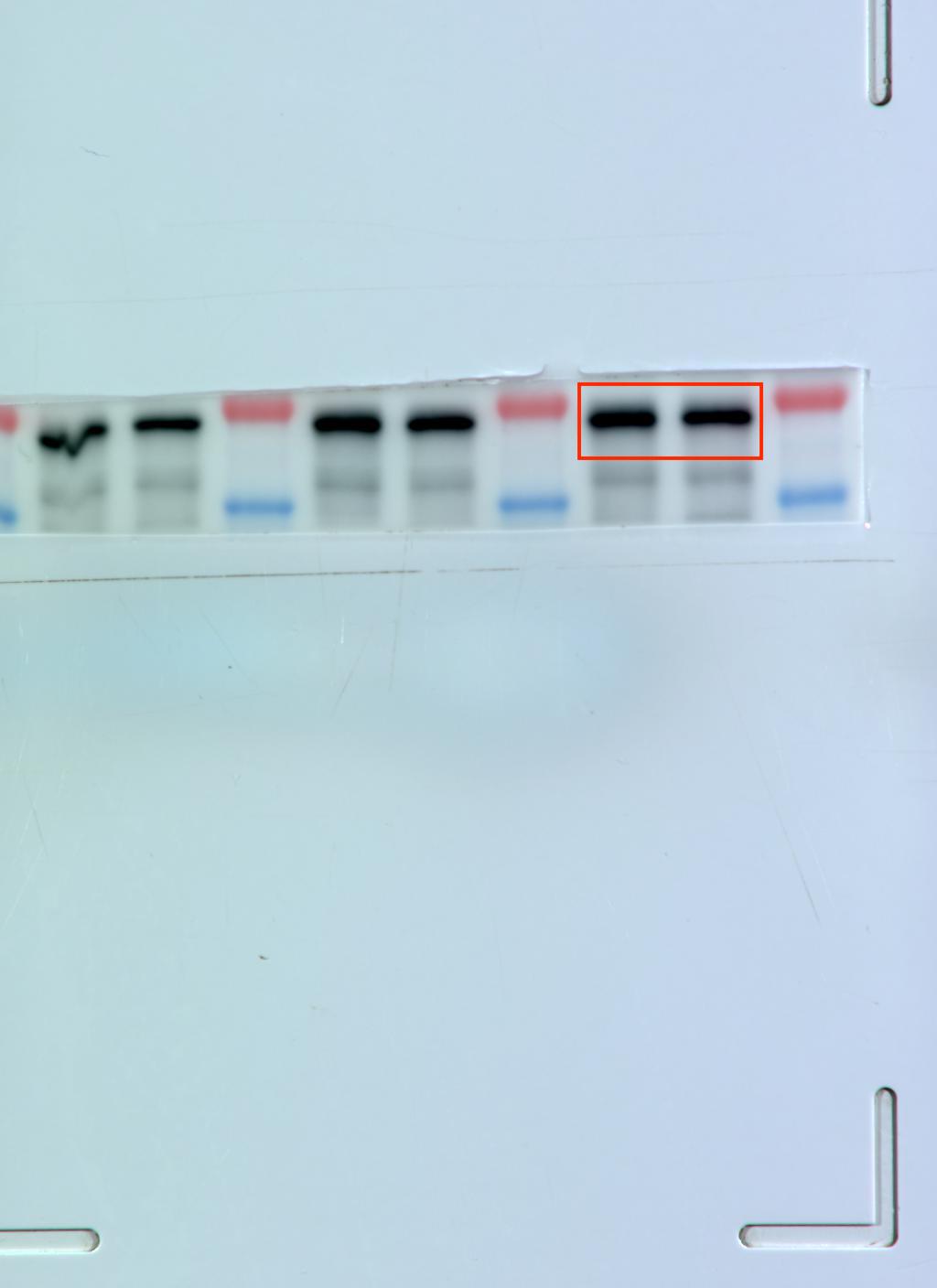

Supplement: Supplementary file 5 [file DataSheet1.zip › Raw data-WB images/Figure 6F/Figure 6F-NFκB p65/Figure 6F-NFκB p65- region.jpg]

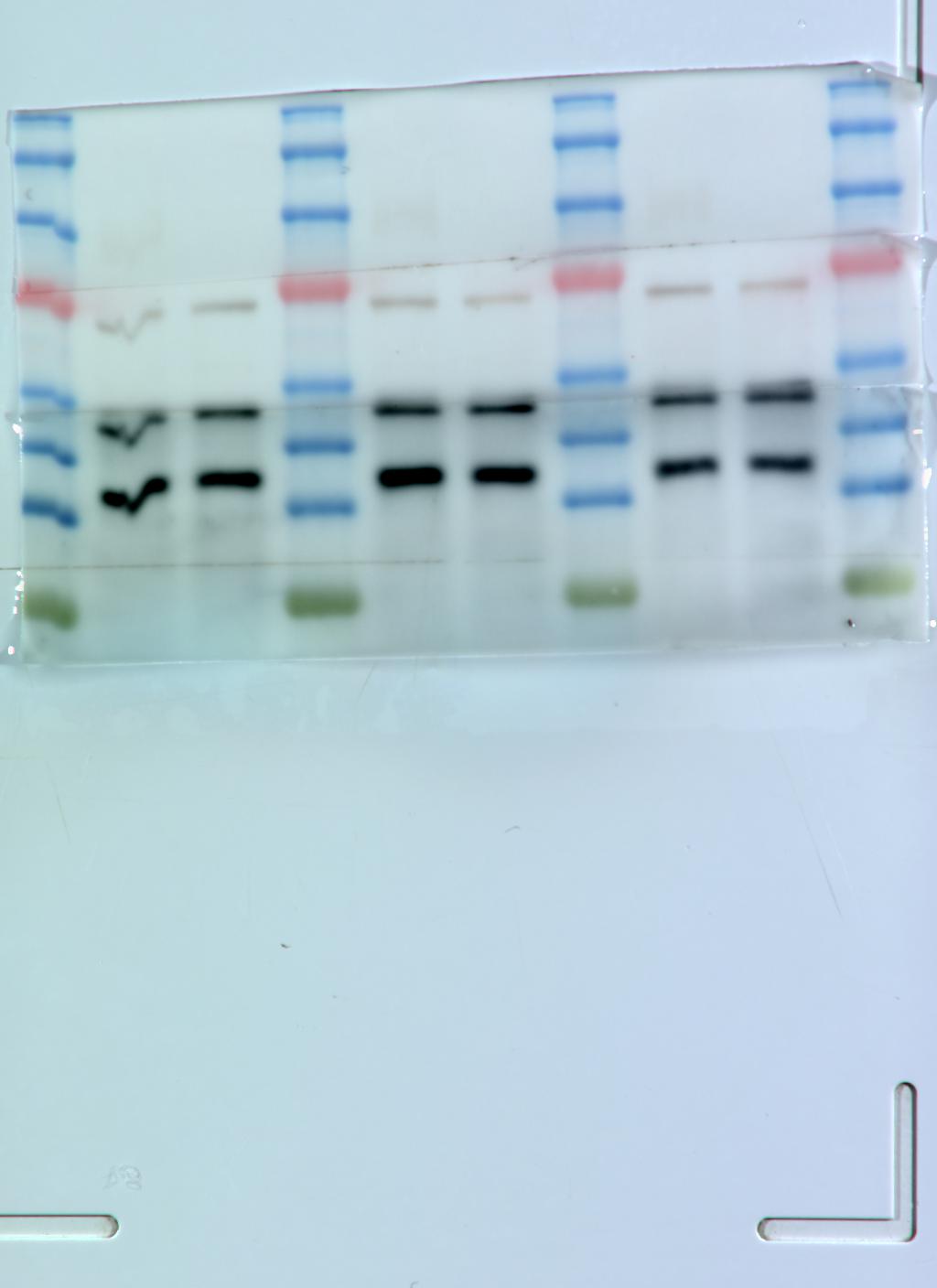

Supplement: Supplementary file 5 [file DataSheet1.zip › Raw data-WB images/Figure 6F/Figure 6F-NFκB p65/Figure 6F-NFκB p65-ALL.jpg]

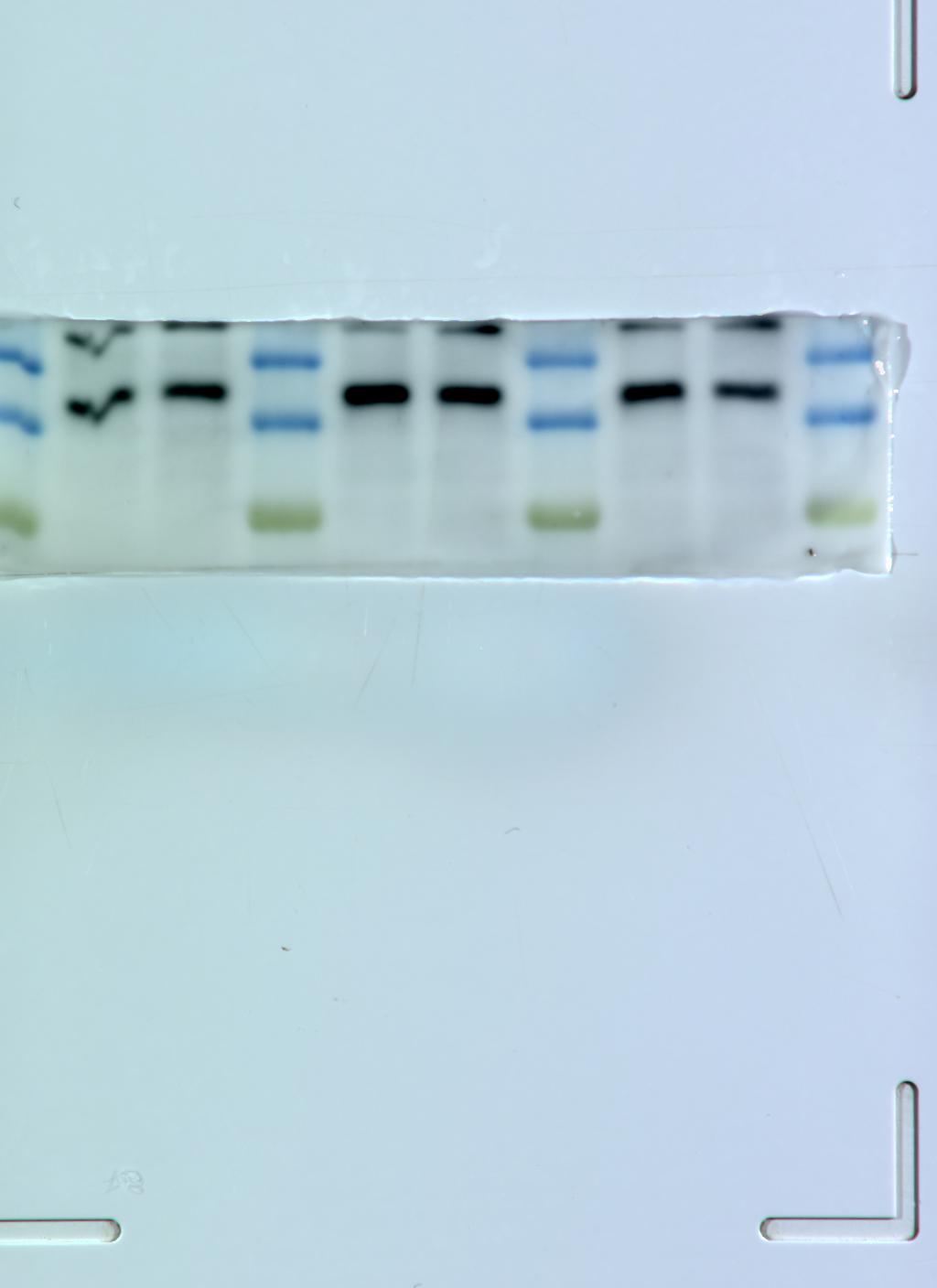

Supplement: Supplementary file 5 [file DataSheet1.zip › Raw data-WB images/Figure 6F/Figure 6F-NFκB p65/Figure 6F-NFκB p65-GAPDH.jpg]

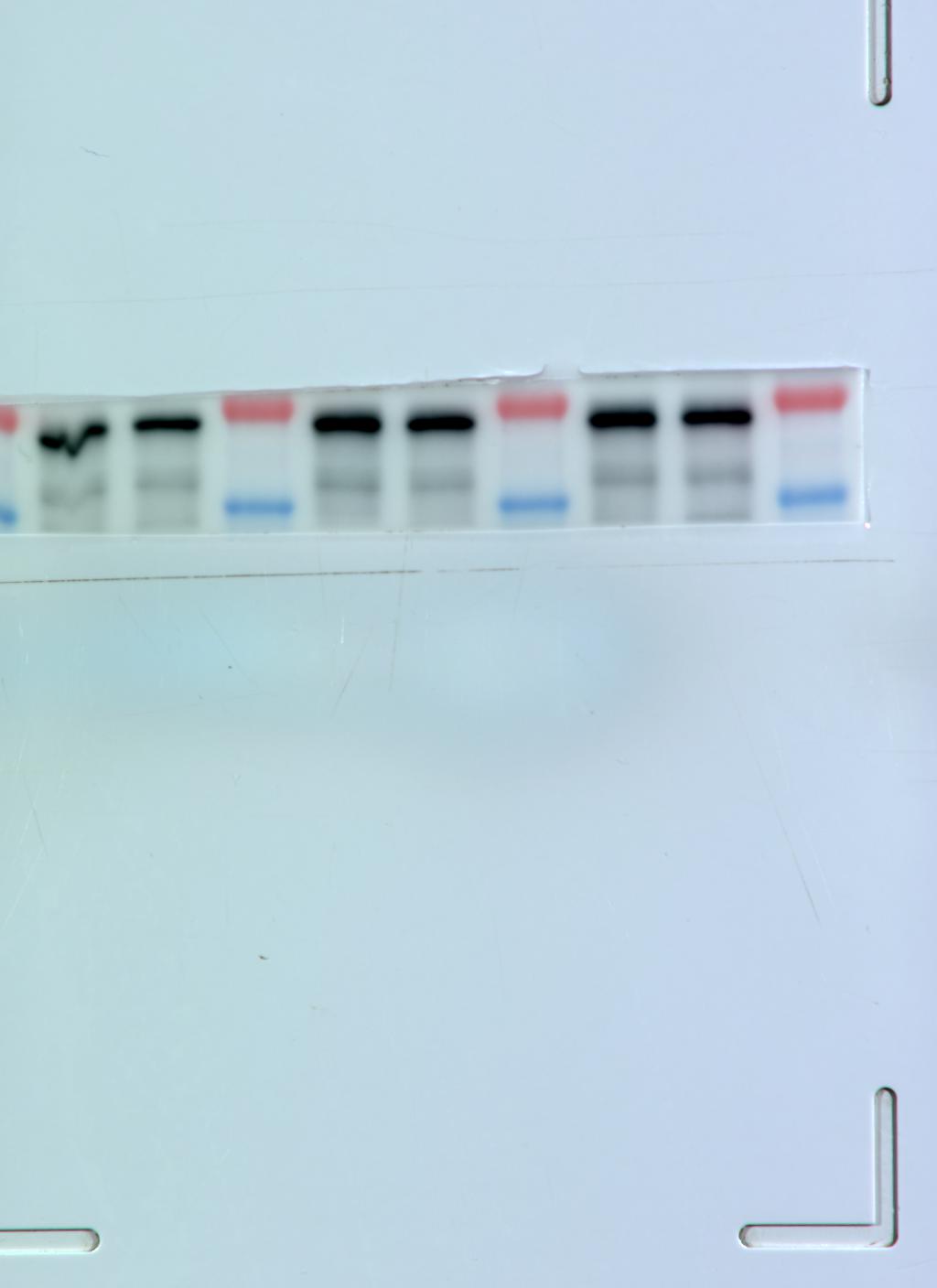

Supplement: Supplementary file 5 [file DataSheet1.zip › Raw data-WB images/Figure 6F/Figure 6F-NFκB p65/Figure 6F-NFκB p65.jpg]

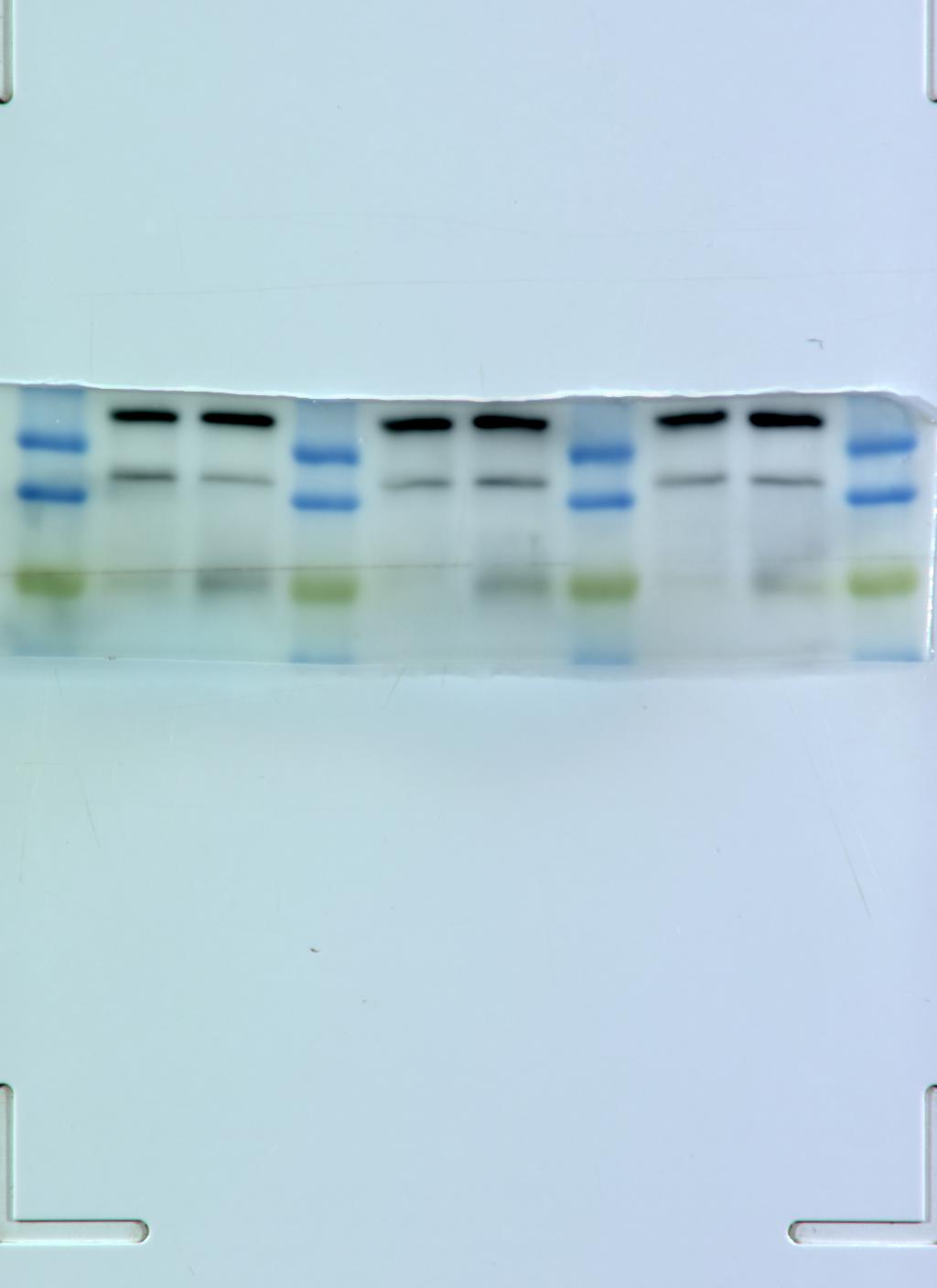

Supplement: Supplementary file 5 [file DataSheet1.zip › Raw data-WB images/Figure 6F/Figure 6F-p-IκBα/Figure 6F-Beita-ACTIN.jpg]

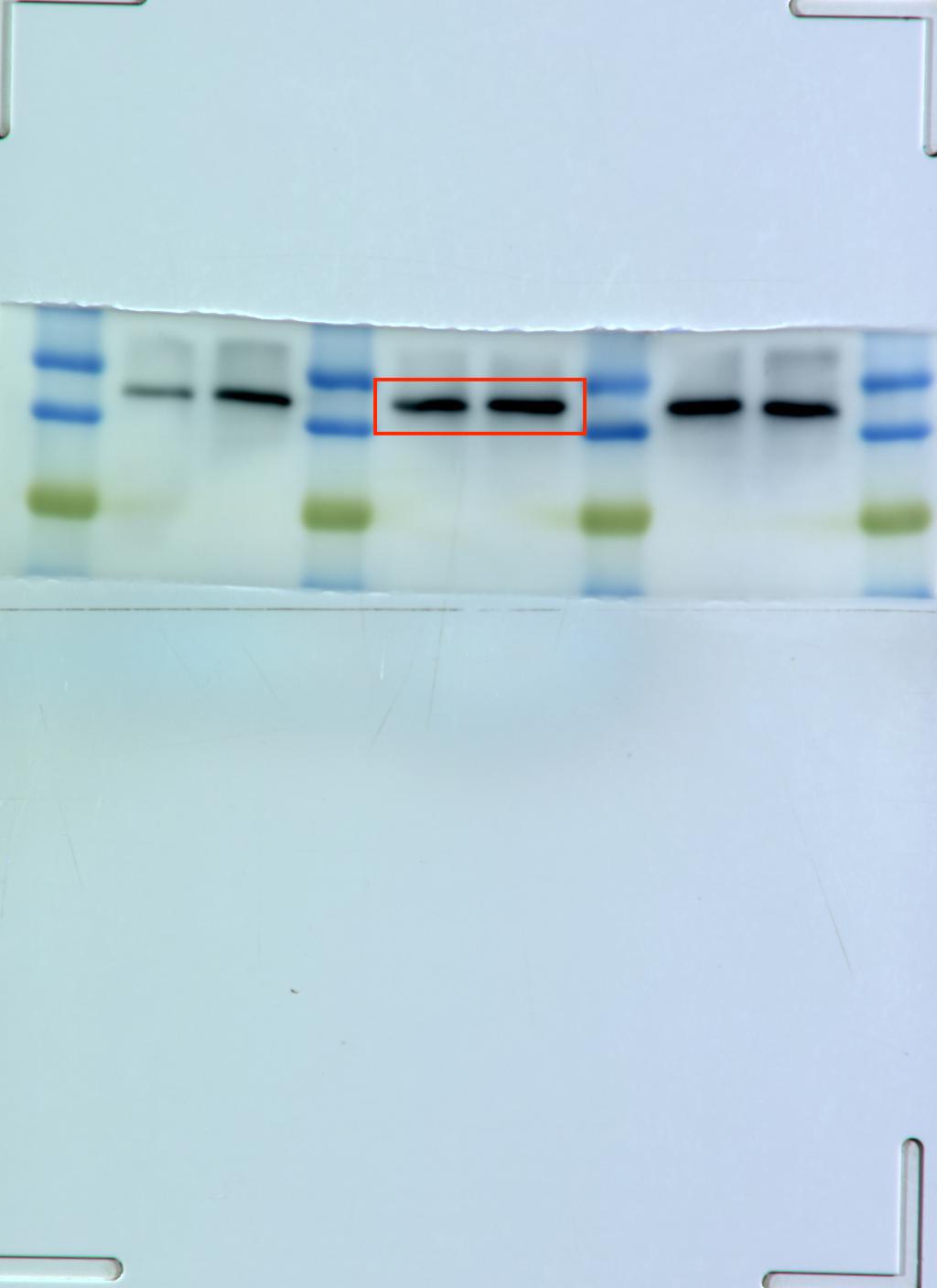

Supplement: Supplementary file 5 [file DataSheet1.zip › Raw data-WB images/Figure 6F/Figure 6F-p-IκBα/Figure 6F-p-IκBα- region.jpg]

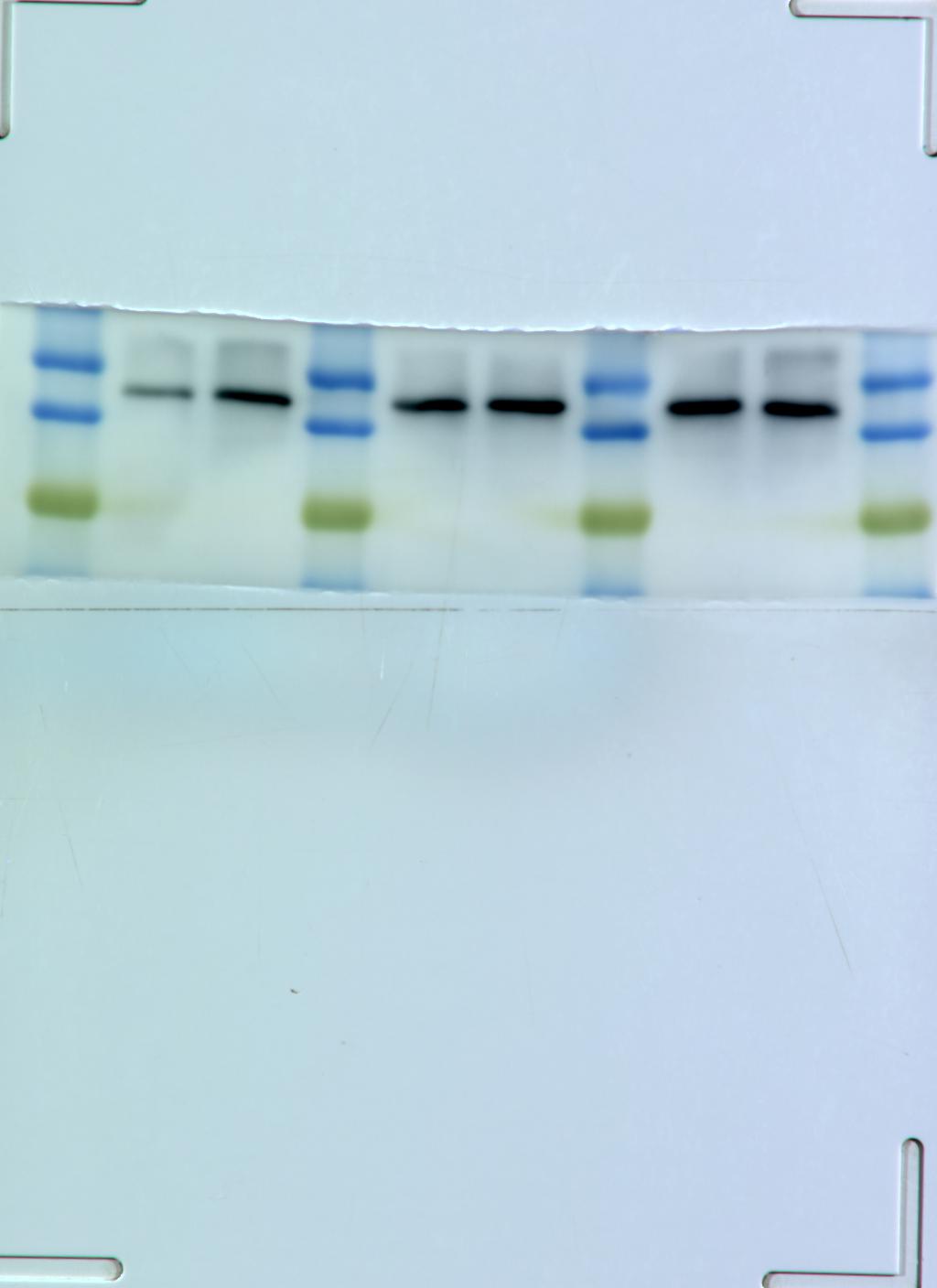

Supplement: Supplementary file 5 [file DataSheet1.zip › Raw data-WB images/Figure 6F/Figure 6F-p-IκBα/Figure 6F-p-IκBα.jpg]

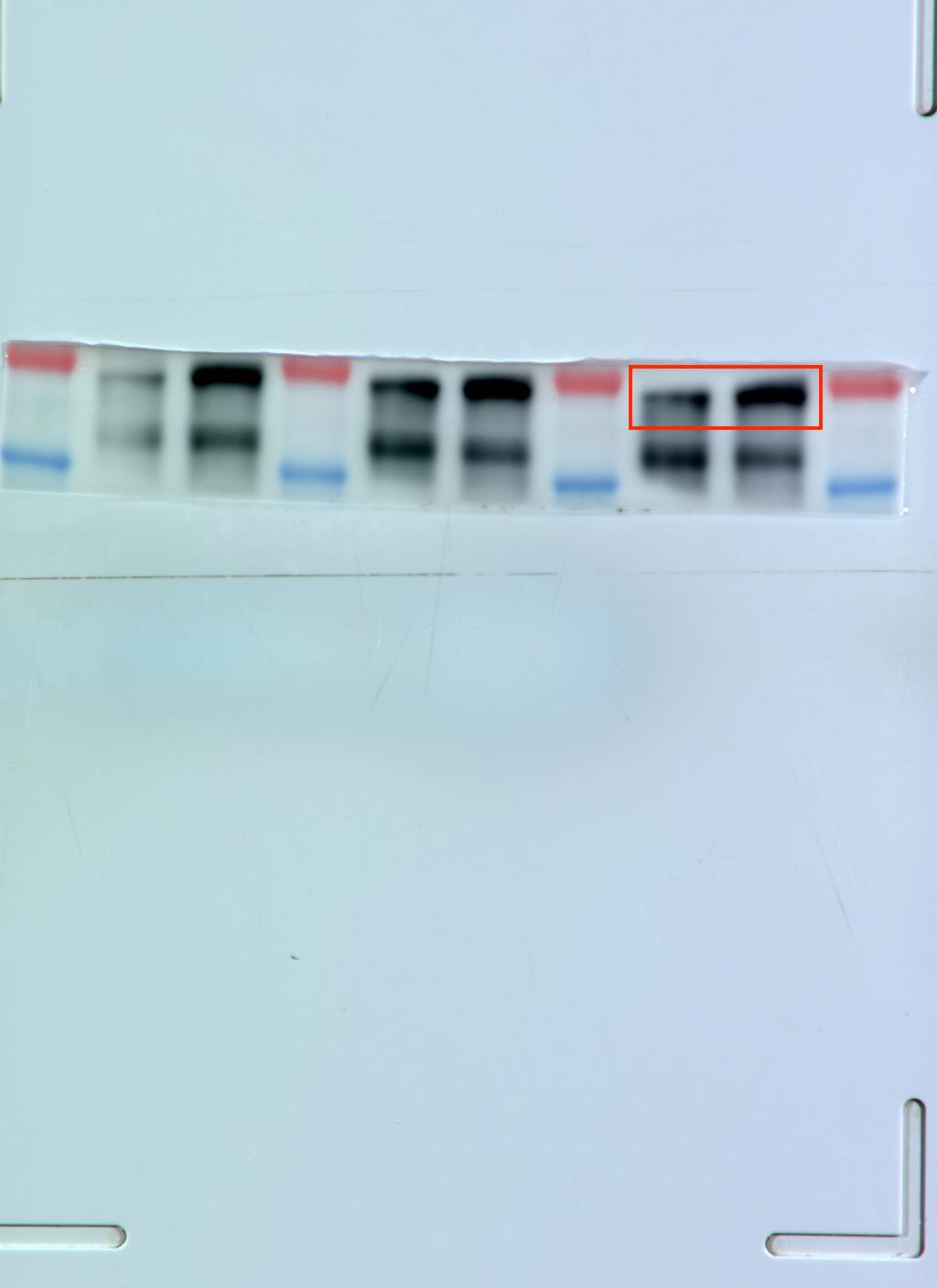

Supplement: Supplementary file 5 [file DataSheet1.zip › Raw data-WB images/Figure 6F/Figure 6F-p-NFκB p65/Figure 6F-p-NFκB p65- region.jpg]

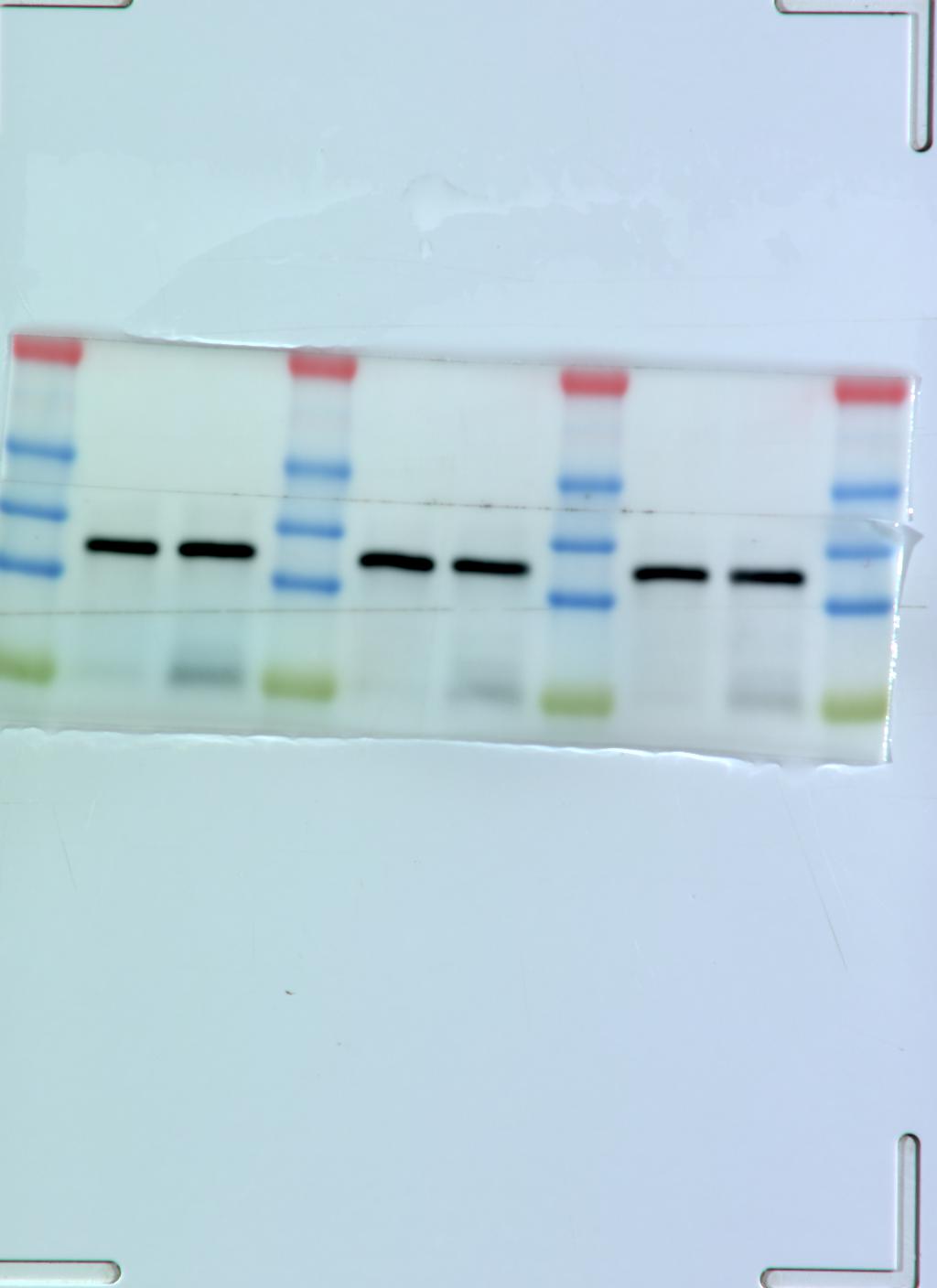

Supplement: Supplementary file 5 [file DataSheet1.zip › Raw data-WB images/Figure 6F/Figure 6F-p-NFκB p65/Figure 6F-p-NFκB p65-ALL.jpg]

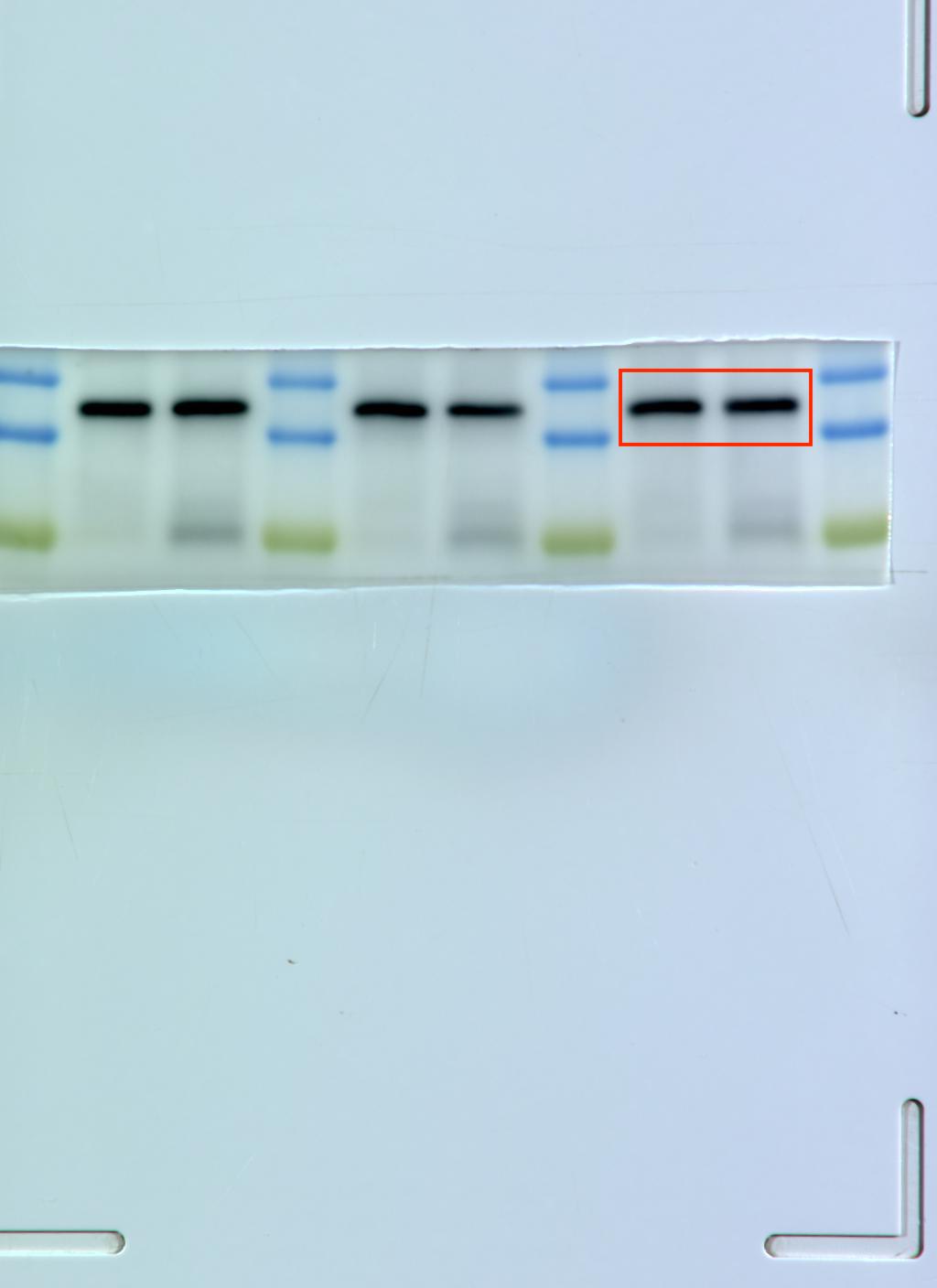

Supplement: Supplementary file 5 [file DataSheet1.zip › Raw data-WB images/Figure 6F/Figure 6F-p-NFκB p65/Figure 6F-p-NFκB p65-GAPDH- region.jpg]

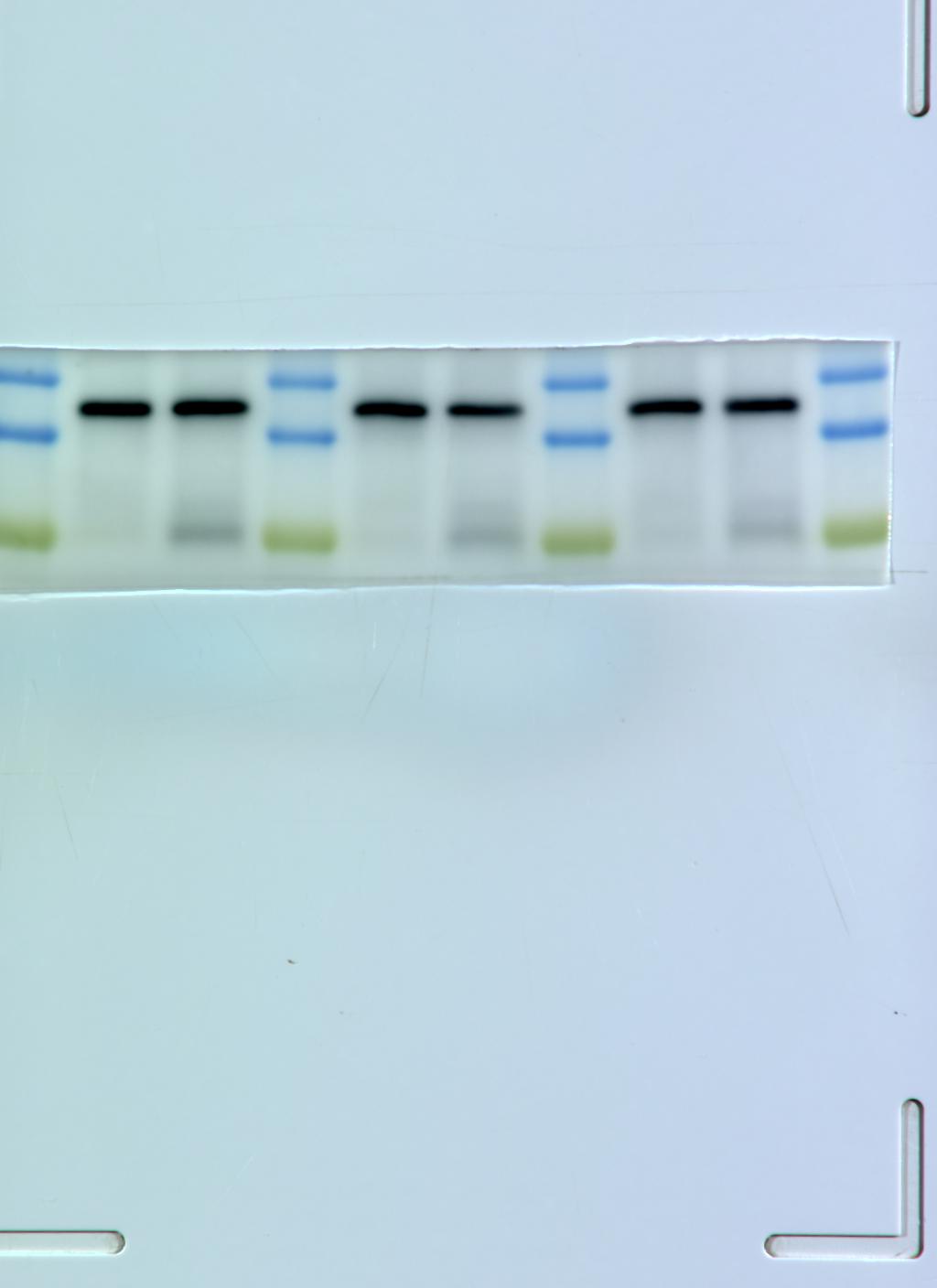

Supplement: Supplementary file 5 [file DataSheet1.zip › Raw data-WB images/Figure 6F/Figure 6F-p-NFκB p65/Figure 6F-p-NFκB p65-GAPDH.jpg]

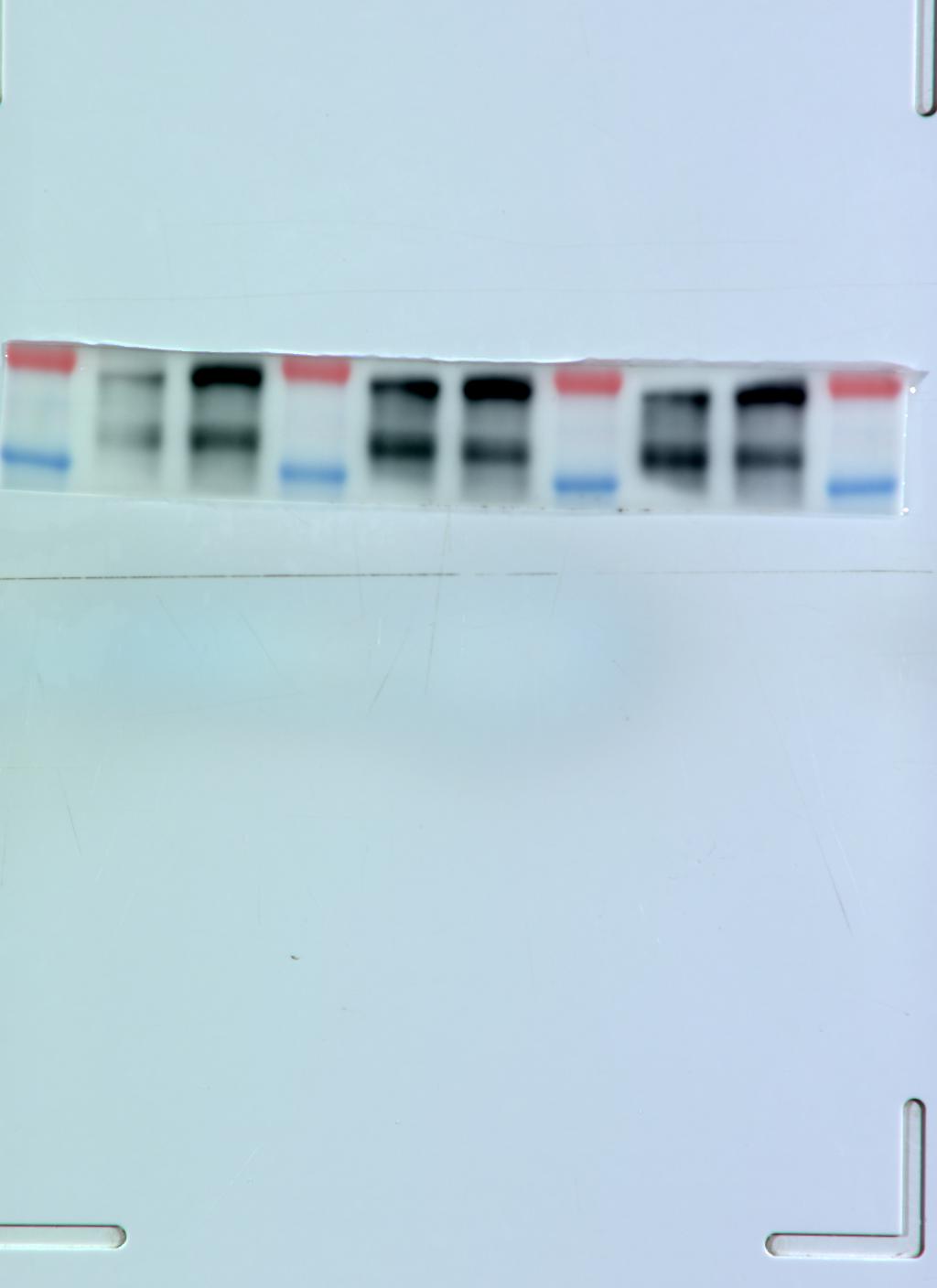

Supplement: Supplementary file 5 [file DataSheet1.zip › Raw data-WB images/Figure 6F/Figure 6F-p-NFκB p65/Figure 6F-p-NFκB p65.jpg]
