# Supplementary material for: Integrin β3 deficiency unleashes spontaneous pulmonary inflammation by promoting B cell hyperactivation via the CD40-CD40L axis
Source: Front Immunol. 2026 Mar 24;17:1796926. doi: 10.3389/fimmu.2026.1796926 (PMC13055533; doi:10.3389/fimmu.2026.1796926)
Supplement: Supplementary file 2 [file Image2.pdf]

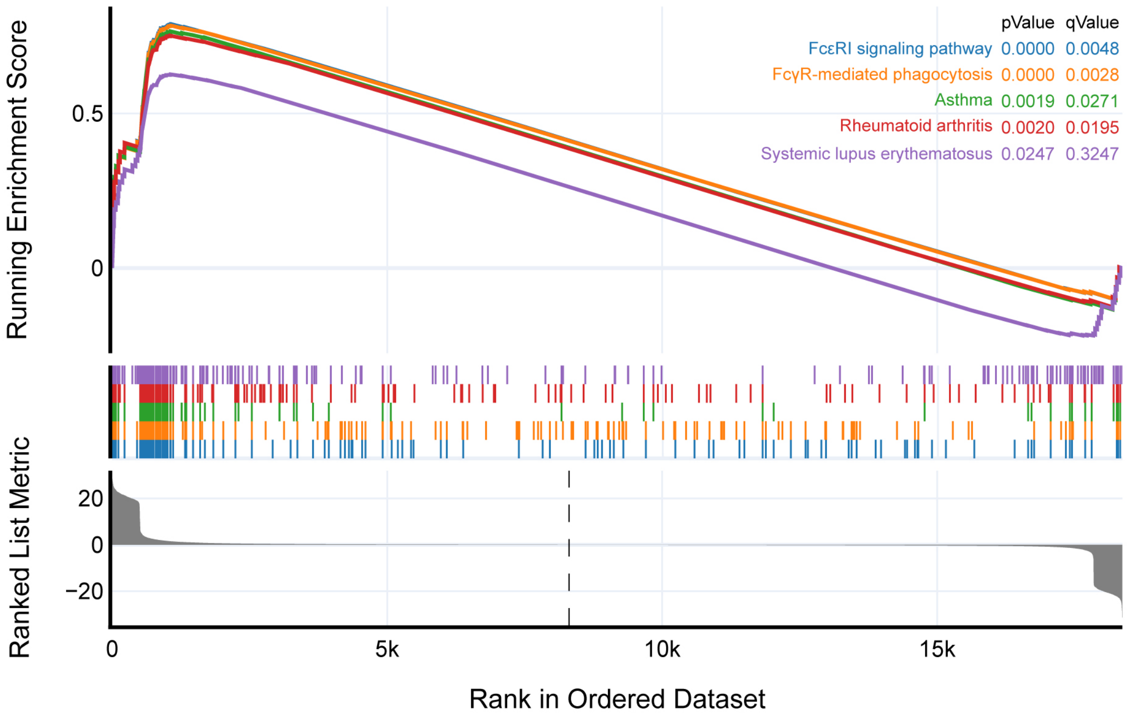

Supplementary Figure 2. Gene Set Enrichment Analysis (GSEA) of KEGG Pathways in lung tissue from WT and β3$^{-/-}$ mice. The plot depicts the running enrichment score (ES) as the analysis walks down the ranked gene list. The peak of the curve corresponds to the maximum ES. Genes belonging to the leading-edge subset are shown as vertical ticks in the middle portion of the plot. The p Value and false discovery rate (FDR) q Value are indicated.
