# Supplementary material for: Integrin β3 deficiency unleashes spontaneous pulmonary inflammation by promoting B cell hyperactivation via the CD40-CD40L axis
Source: Front Immunol. 2026 Mar 24;17:1796926. doi: 10.3389/fimmu.2026.1796926 (PMC13055533; doi:10.3389/fimmu.2026.1796926)
Supplement: Supplementary file 3 [file Image3.pdf]

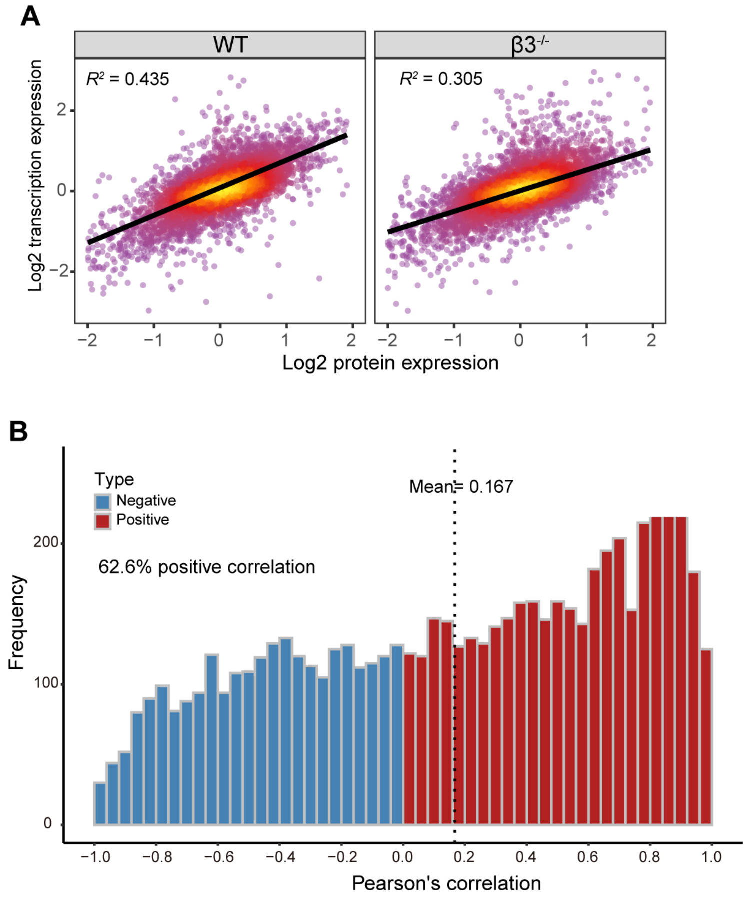

Supplementary Figure 3. Proteomic profiling and integrated transcriptomic-proteomic analysis of β3$^{-/-}$ splenic tissues. (A) Scatter plot comparing transcriptomic (RNA-seq log2 fold change) and proteomic (protein abundance log2 fold change) alterations for matched genes/proteins between β3-/- and WT spleen. The coefficient of determination (R^2) from linear regression is indicated. (B) Cumulative distribution plots of Pearson correlation coefficients between matched transcript and protein expression levels across all samples within each genotype. The x-axis represents the Pearson correlation coefficient (r), and the y-axis represents the cumulative proportion of gene-protein pairs. Curves are colored based on the direction of correlation: red for positive correlations ($r > 0$) and blue for negative correlations ($r < 0$).
