# Supplementary material for: Integrin β3 deficiency unleashes spontaneous pulmonary inflammation by promoting B cell hyperactivation via the CD40-CD40L axis
Source: Front Immunol. 2026 Mar 24;17:1796926. doi: 10.3389/fimmu.2026.1796926 (PMC13055533; doi:10.3389/fimmu.2026.1796926)
Supplement: Supplementary file 4 [file Image4.pdf]

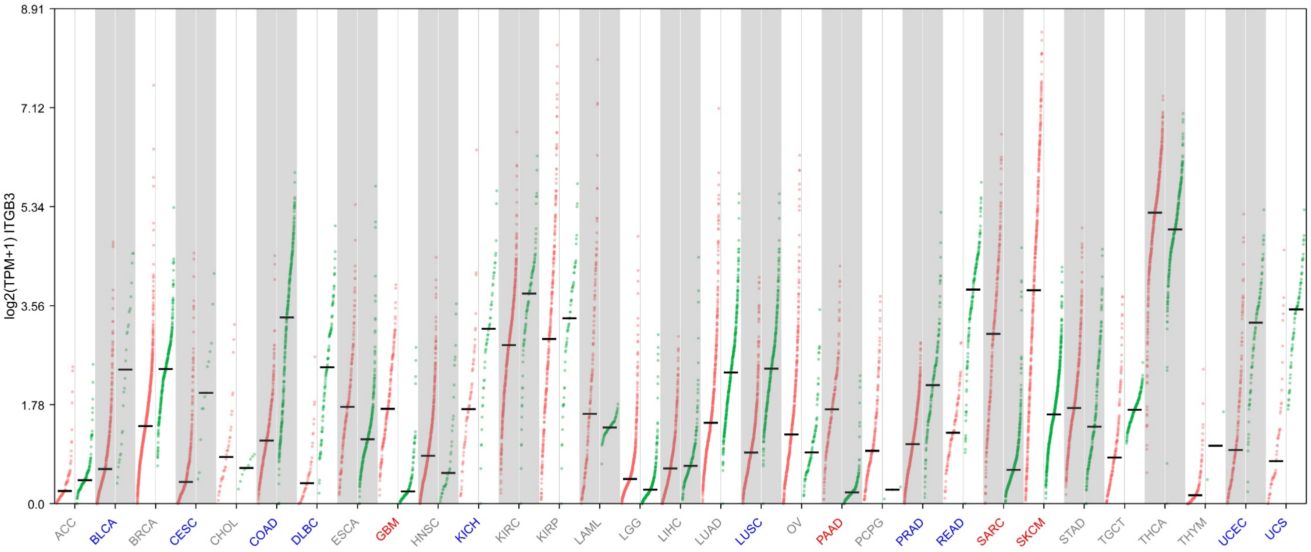

Supplementary Figure 4. Pan-cancer expression landscape of *ITGB3* in TCGA tumors and matched normal tissues. The gene expression profile of *ITGB3* across 33 human cancer types was analyzed using the GEPIA web server. The scatter plot displays the log2 (TPM+1) expression levels in tumor tissues (red dots) compared to normal tissues (green dots). Each dot represents an individual sample. The x-axis indicates the standard TCGA cancer type abbreviations.
