# Supplementary material for: Integrin β3 deficiency unleashes spontaneous pulmonary inflammation by promoting B cell hyperactivation via the CD40-CD40L axis
Source: Front Immunol. 2026 Mar 24;17:1796926. doi: 10.3389/fimmu.2026.1796926 (PMC13055533; doi:10.3389/fimmu.2026.1796926)

Figure 1A

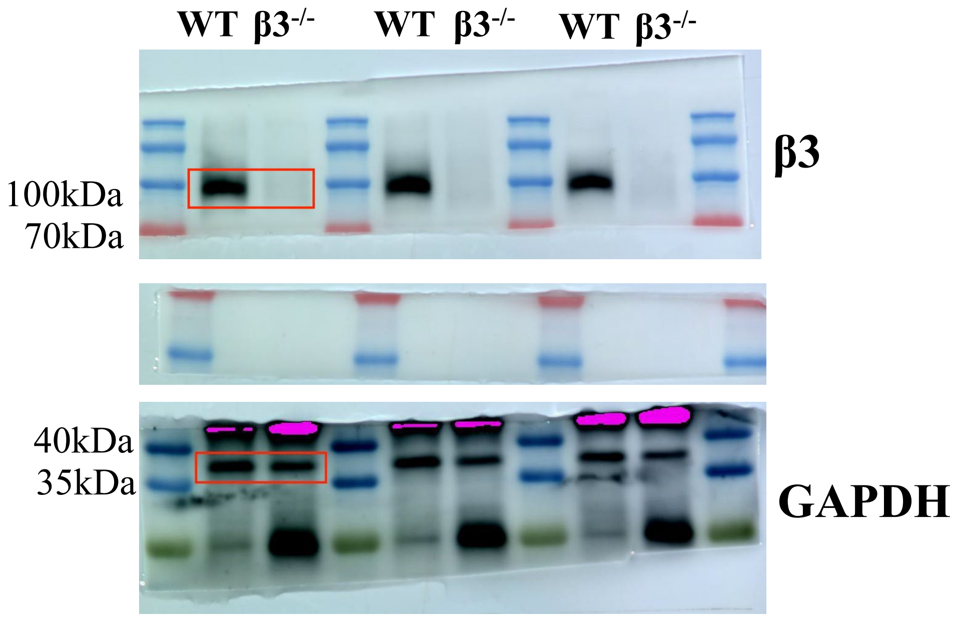

All membrane with marker

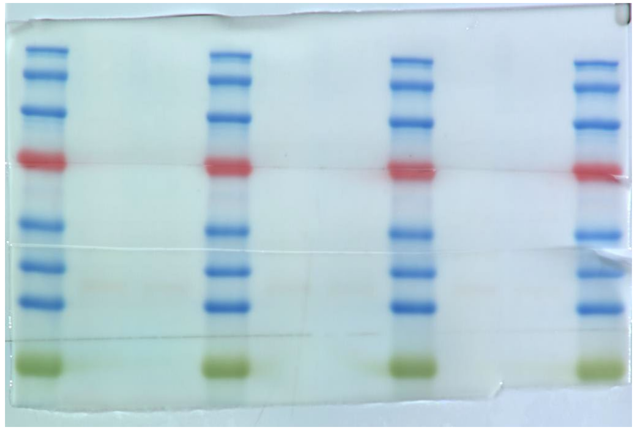

Figure 6A

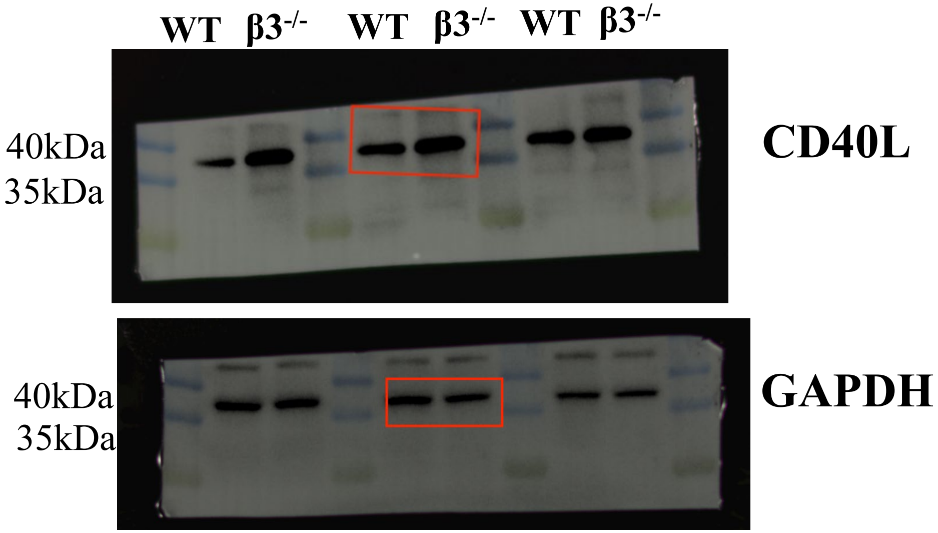

Figure 6F-IκBα

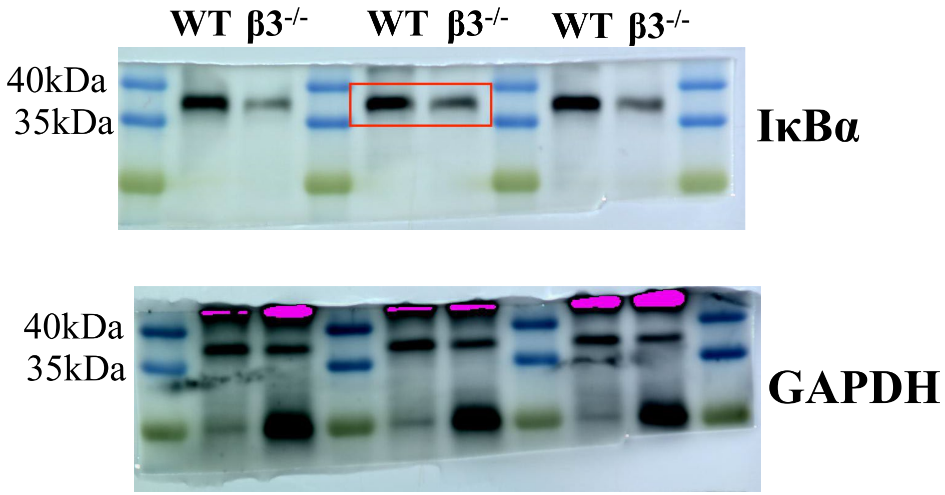

Figure 6F-p-IκBα

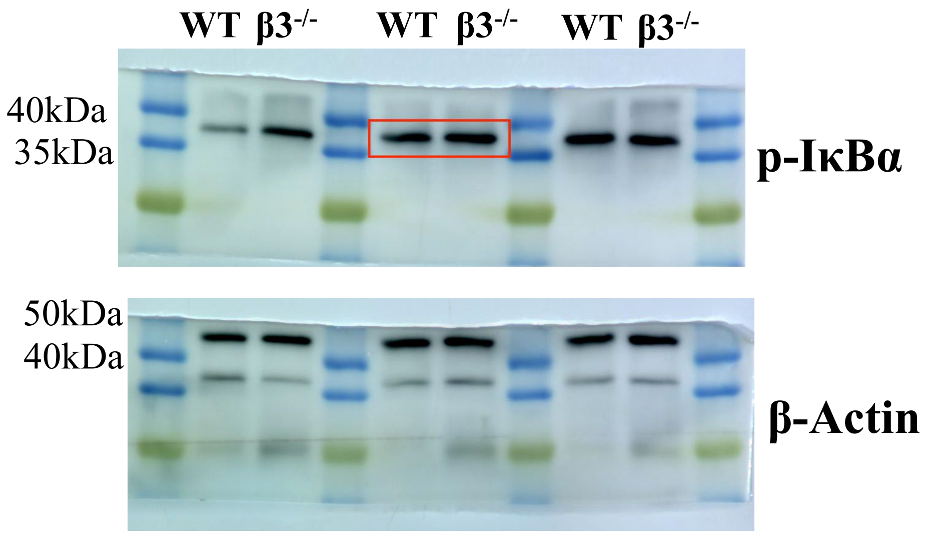

Figure 6F-NFκB p65

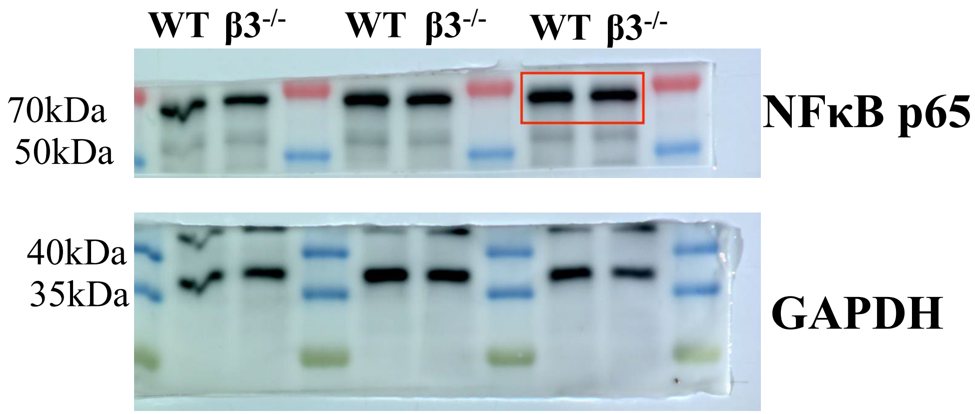

All membrane with marker

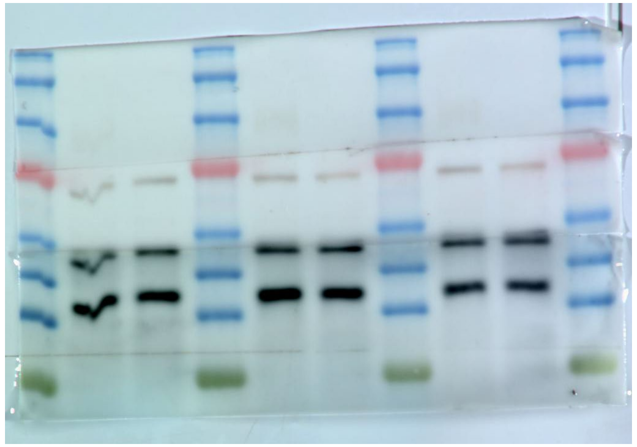

Figure 6F-p-NFκB p65

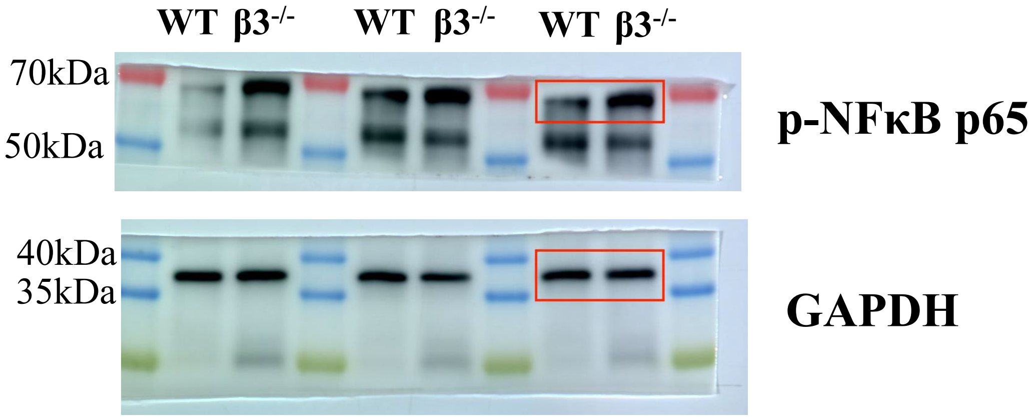

All membrane with marker

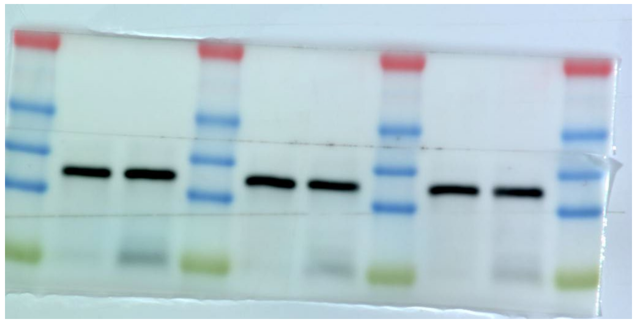

Supplement: Supplementary file 5 [file DataSheet1.zip › Raw data-WB images/Assembled Original Blots.pdf]
